# Gene expansion in the hawkmoth *Manduca sexta* drives evolution of food-associated odorant receptors

**DOI:** 10.1016/j.isci.2024.111317

**Published:** 2024-11-04

**Authors:** Megha Treesa Tom, Philipp Brand, Sascha Bucks, Jin Zhang, Mario Ernesto Escobar Huezo, Bill S. Hansson, Sonja Bisch-Knaden

**Affiliations:** 1 Department of Evolutionary Neuroethology, Max-Planck Institute for Chemical Ecology, Jena, Germany; 2 Laboratory of Neurophysiology and Behavior, The Rockefeller University, New York, NY, USA; 3 Department of Entomology, College of Plant Protection, Nanjing Agricultural University, Nanjing, China

**Keywords:** Sensory neuroscience, Evolutionary ecology, Cell biology

## Abstract

In insects, odorant receptors (ORs) are required for the detection of most olfactory cues. We investigated the function of a clade of four duplicated *ORs* in the hawkmoth *Manduca sexta* and found that these paralogs encode broadly tuned receptors with overlapping but distinct response spectra. Two paralogs, which arose after divergence from a related lineage, show high sensitivity to floral esters released by a nectar-rich plant frequently visited by *M. sexta*. Functional imaging in mutant moths lacking one of the paralogs suggests that olfactory sensory neurons expressing this OR target a previously identified feeding-associated glomerulus in the primary olfactory center of the brain. However, only the response of this glomerulus to the single ligand unique to the now mutated OR disappeared, suggesting neuronal coexpression of the paralogs. Our results suggest a link between the studied *OR* expansion and enhanced detection of odors emitted by valuable nectar sources in *M. sexta*.

## Highlights

• Gene duplication gave rise to a clade of hawkmoth odorant receptors
• These paralogous receptors have broad and overlapping but distinct response profiles
• Two paralogs are most sensitive to rare floral volatiles from a valuable nectar source
• The paralogs are expressed in neurons that target a food-associated brain region

## Graphical abstract

**Figure:**
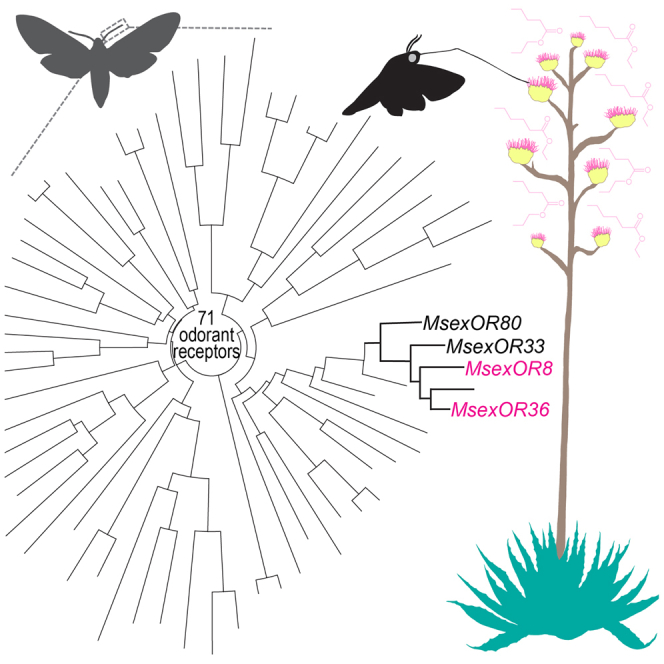

## Introduction

Insects can detect and discriminate a wide variety of odors that help them filter their chemosensory environment to find food and mates, rear their offspring, and avoid parasites, competitors, and toxic substances.^1^ This olfactory ability is conferred by a large repertoire of olfactory receptors expressed in olfactory sensory neurons (OSNs) that innervate several sensory organs, primarily the antennae. Olfactory receptors mostly belong to the gene family of odorant receptors (ORs), which are capable of binding ligands from many chemical classes and are thus responsible for detecting the majority of ecologically important cues.^2^^,^^3^^,^^4^^,^^5^^,^^6^ A functional receptor is a heteromeric complex consisting of multiple subunits of the conserved “odorant receptor co-receptor” ORCo and an odor-selective OR.^7^ The spectra of individual ORs can range from narrowly tuned to broadly tuned.^4^ A narrowly tuned OR binds only one or a few compounds that often belong to the same chemical class, such as pheromones or odors that signal harmful sources, whereas broadly tuned ORs have lower specificity and detect odors from several chemical classes, such as odors emitted by plants or fruits.^8^ Through combinatorial odor coding, the broadly tuned or generalist ORs allow the olfactory system to detect and discriminate between hundreds and thousands of volatiles.^9^ Combinatorial coding appears to be particularly important for the perception of natural odor mixtures, such as the headspace of flowers or host plants.^10^

The size of the *OR* repertoire varies between insect species, with only five genes in the jumping bristletail *Machilis hrabei* and ∼500 in the clonal raider ant *Ooceraea biroi*.^11^^,^^12^ This variability is due to independent gains and losses of *OR* genes along different lineages through a process of gene “birth and death.”^13^^,^^14^^,^^15^ Both gain and loss of *OR* genes have been associated with chemosensory adaptation in insects. Host specialization, endemism, parasitism, etc. often correlate with contraction of the *OR* gene repertoire,^16^^,^^17^^,^^18^^,^^19^^,^^20^ while the expansion of *OR* clades has been associated with the extension of chemosensory capabilities to ecologically important cues such as pheromones or host volatiles.^21^^,^^22^^,^^23^ Accordingly, the evolution of novel *ORs* is critical for the diversification of insect lineages.

*ORs* are mainly maintained under purifying selection,^17^^,^^24^ but when a gene duplicates, relaxed selection on the redundant gene copies allows an increased accumulation of mutations. Many of these mutations can cause pseudogenization, rendering the gene non-functional and leading to its death. However, mutations at critical amino acid positions can lead to changes in receptor function that, if beneficial, may be positively selected for, resulting in the eventual birth of a new gene. Such duplicates follow one of two main paths of functional evolution—subfunctionalization or neofunctionalization—where the former refers to the loss of a subset of the ancestral function of each duplicate so that their joint function is that of the ancestral gene, and the latter to the evolution of a new function by at least one duplicate.^25^ Subfunctionalization can precede neofunctionalization,^26^ but both mechanisms can also occur simultaneously.^27^ In the case of ORs, the binding of an odorant is considered a function, and thus subfunctionalization and neofunctionalization are a result of the change in odorant-binding capabilities of the diverging gene copies.^13^^,^^22^

The hawkmoth *Manduca sexta* is a large nocturnal insect that feeds on floral nectar while hovering in front of a flower. Mutagenesis of ORCo revealed that feeding behavior is completely dependent on the presence of functional ORs.^28^ *M. sexta* harbors a repertoire of approximately 70 ORs in its genome,^29^ few of which have been functionally characterized.^30^^,^^31^^,^^32^^,^^33^ A phylogenetic analysis with six other Lepidopteran species^29^ identified an *M. sexta*-specific clade of five previously uncharacterized ORs. The corresponding genes are clustered in the same tandem array in the genome, and all but one OR have intact gene structures. This clade had a single, ester-tuned orthologous OR in *Bombyx mori* (BmorOR24^34^) in the phylogenetic analysis. Because orthologous ORs often have similar response profiles,^30^^,^^35^ we hypothesized that the paralogous ORs of *M. sexta* might also respond to esters, a chemical class known to reliably induce feeding behavior in wind tunnel experiments with female hawkmoths.^36^

We aimed at de-orphanizing this clade of paralogous ORs by ectopically expressing them in the *Drosophila melanogaster* empty neuron system,^37^ a method that has been widely used for functional characterization of insect ORs.^2^^,^^4^^,^^38^^,^^39^ To this end, we screened the *M. sexta* ORs against a large panel of chemically diverse odorants with known behavioral relevance and odor-evoked activation patterns in the brain’s first olfactory processing center, the antennal lobe.^36^ This, in turn, allowed us to link OR response profiles to those of olfactory glomeruli, which are anatomical and functional subunits of the antennal lobe, each receiving input from OSNs that typically express a unique odor-selective OR.^40^^,^^41^ Therefore, we sought to identify putative glomerular targets of the investigated ORs and to validate the results by knock-out experiments. By comparing receptor binding profiles with those of glomerular activation levels previously associated with specific behaviors,^36^ we aimed to gain insight into the functional significance of this OR expansion in *M. sexta*.

## Results and discussion

### Paralogous MsexORs have broad and overlapping but distinct response profiles

**Figure 1:**
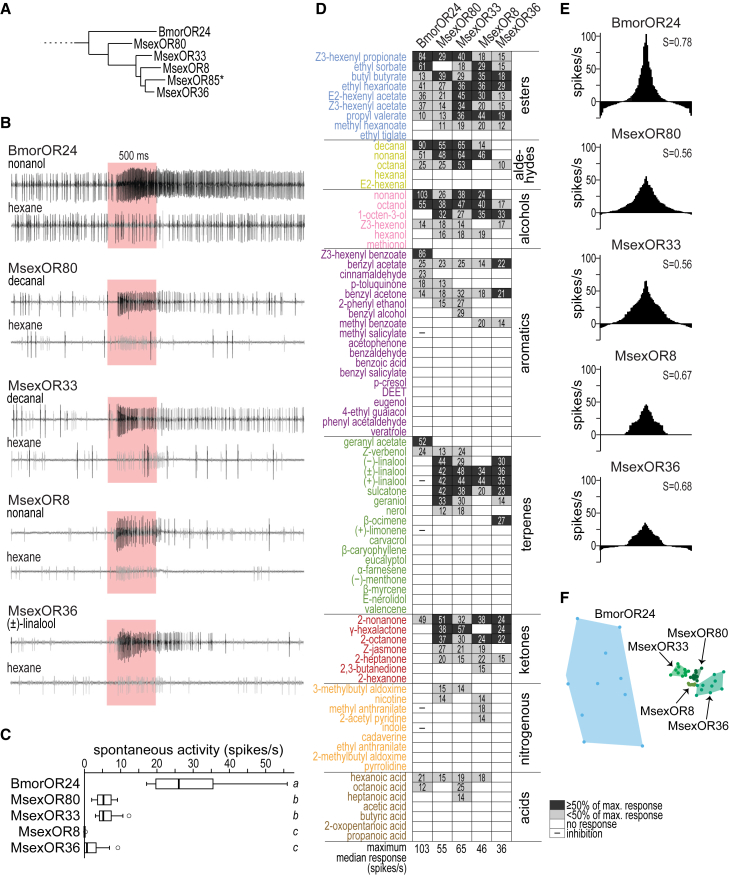
Paralogous MsexORs have broad and overlapping but distinct response profiles Odor responses of the four intact paralogous MsexORs and their common ortholog BmorOR24 ectopically expressed in the *D. melanogaster* empty neuron system (deletion mutant (*Δhalo*) lacking endogenous OR genes in the A neuron of ab3 sensilla). (A) Branch of the studied clade of MsexORs and their orthologous OR in *B. mori*, from a published phylogenetic tree that includes seven Lepidopteran species.^29^*Asterisk*, pseudogene. (B) Representative single sensillum recordings from each OR to odors that induced the maximum median solvent-subtracted response together with the corresponding solvent (hexane) response from the same fly. *Large black spikes*, A neuron; *small gray spikes*, B neuron; *red background*, stimulus duration. See also Figure S1. (C) Spontaneous activity of ab3 A neurons of test flies *Δhalo*; *DmelOr22a-Gal4*/*UAS-ORX* expressing moth ORs (*n* = 18–34). *Boxplots*, median, interquartile range and range; *empty circles*, outliers; *different letters next to boxplots*, *p* < 0.05, Kruskal-Wallis Test with Dunn’s multiple comparisons test. (D) Response profiles of moth ORs to 80 odors (Table S1) at a dilution of 10^−2^. *Numbers in cells*, median solvent-subtracted responses; *dark gray cells*, ≥ half maximum median response of the OR; *light gray cells*, < half maximum response; *white cells*, no response (<10 spikes/s); *−*, inhibitory responses in BmorOR24 (spontaneous activities in MsexORs was too low to calculate inhibition; see C); sample size: *n* = 9–10 (BmorOR24), *n* = 9–11 (MsexOR80, 33, 8), *n* = 8–10 (MsexOR36); odors sorted by chemical class. (E) Tuning curves based on median responses (y axis) to 80 odors (x axis). S, lifetime sparseness value (calculated as in a study by Bhandawat et al.^42^); values range from S = 0 (equal response to all odors tested) to S = 1 (response to only one odor). See also Figure S2. (F) Non-metric multidimensional scaling plot (Euclidean, 2D stress: 0.13). Each dot represents responses (*n* = 8–11/OR) to 80 odors. See Table S6.

To investigate the function of the four intact paralogous *M. sexta* ORs (Figure 1A), we expressed individual moth ORs in the *D. melanogaster* “empty neuron” system, i.e., mutant antennal neurons (A neuron of the fly ab3 sensilla) that lack endogenous ORs.^37^ The *B. mori* ortholog BmorOR24 was previously studied using a different expression system and a smaller odorant set.^34^ For a better comparison with its *M. sexta* orthologs, we also expressed BmorOR24 in the fly empty neuron system. We recorded odor-evoked responses of these moth ORs to 80 ecologically relevant and chemically diverse odorants using the single sensillum recording (SSR) technique. As a control, we tested “empty” A neurons without OR expression and found no clear response, but occasionally bursts of spikes upon stimulation with odors that strongly activated the neighboring B neuron (Figure S1). In contrast, when a moth OR was expressed, we observed clear odor-evoked A neuron responses (Figure 1B). The spontaneous activity of the neuron depended on the ectopically expressed OR, as observed in *D. melanogaster*,^43^ but was similar between MsexOR80 and 33 and between MsexOR8 and 36 (Figure 1C).

Each of the four MsexORs and BmorOR24 responded to many of the tested odorants (29%–45%, Figure 1D), demonstrating that none of these ORs seems to be a specialized receptor. In particular, MsexOR80 and 33 (lifetime sparseness S = 0.56, Figure 1E) appeared to be relatively broadly tuned compared to other insect ORs of known function (Figure S2). Consistent with a previous report,^34^ aliphatic esters were among the best ligands for BmorOR24, and for the *M. sexta* paralogs we also observed strong activation upon stimulation with esters (Figure 1D). However, each moth OR also responded strongly to odors belonging to other chemical classes, such as aldehydes, alcohols, and aromatics. Twenty of the 23 odors that activated BmorOR24 also activated at least one of the *M. sexta* ORs, and the MsexORs together recognized 23 additional odors. Among these additional ligands, terpenes such as linalool and sulcatone induced particularly strong activation of most MsexORs.

The only partially overlapping response profiles of the MsexOR duplicates suggest that some odorant-binding properties were retained after each gene duplication event, while others were lost, supporting subfunctionalization. However, each OR also had at least one unique ligand, such as β-ocimene, which was detected only by MsexOR36. This presence of unique ligands suggests neofunctionalization, and an analysis of similarities (ANOSIM) shows that each tested moth OR of the clade has a distinct profile (Figure 1F, R = 0.59, *p* < 0.0001, Euclidean distance). Taken together, our data suggest that functions such as responses to esters, aldehydes, and alcohols may be ancestral because they are shared between *M. sexta* paralogs and their silkmoth ortholog, while new functions such as sensitivity to terpenes have also been acquired. Thus, both subfunctionalization and neofunctionalization seem to be evident in the studied clade of paralogous MsexORs.

### Narrow tuning to aldehydes or esters at lower odor concentrations

**Figure 2:**
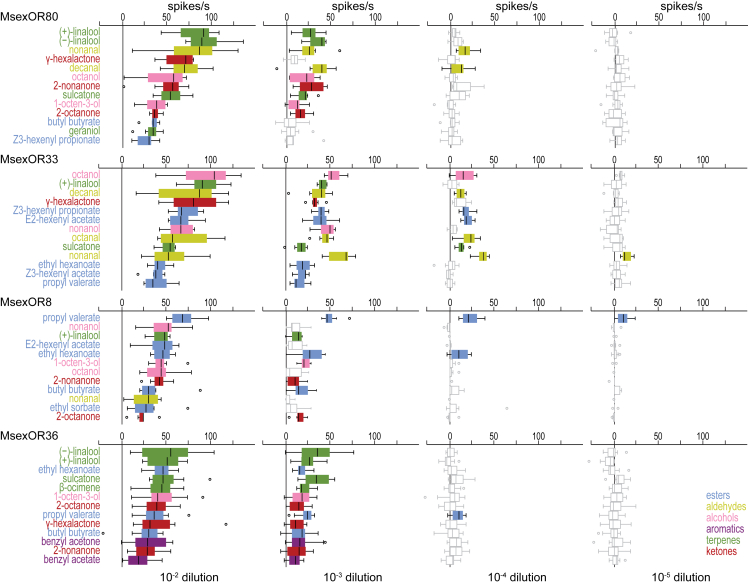
Narrow tuning to aldehydes or esters at lower odor concentrations Dose-response experiments with the best ligands of the four paralogous MsexORs. *Boxplots*, median, interquartile range and range; *circles*, outliers; *colored boxes*, median responses ≥10 spikes/s, *n* = 8–12 for MsexOR36, *n* = 7–8 for remaining ORs. One outlier not shown (MsexOR36 response to butyl butyrate at 10^−2^, 142 spikes/s). Odors are sorted by the median response induced in each of the ORs at a dilution of 10^−2^. (+)- and (−)-linalool were tested individually but racemic (±)-linalool was excluded from the dose-response assay. None of the ORs responded to 10^−6^ and 10^−7^ dilutions of their respective test panels. See Table S7.

When screening the MsexORs with odorants at a dilution of 10^−2^, we found responses to odors from most (MsexOR36) or all (MsexOR80, 33, 8) chemical classes tested (Figure 1D). To assess how the odor specificity of MsexORs changes at lower odor concentrations, we tested dilutions of their respective best ligands (≥ half the maximum median response of a given OR, dark gray cells in Figure 1D), spanning six orders of magnitude (Figure 2). As expected from results obtained in insects^2^^,^^4^ and mammals,^44^ the receptive range of the MsexORs studied narrowed at lower odorant doses. At a dilution of 10^−4^, a 100-fold lower dose than used in the screen (Figure 1D), MsexOR33 was the only receptor that still had a relatively broad response spectrum, being activated by seven odors belonging to four chemical classes, while the other ORs were narrowly tuned to either aldehydes (MsexOR80) or esters (MsexOR8 and 36). At a dilution of 10^−5^, only two of the receptors were activated, each responding to only one odorant: MsexOR33 (nonanal) and MsexOR8 (propyl valerate). Thus, experiments with low odorant concentrations confirmed the functional differences between the closely related MsexORs, which seem to fall into two groups, tuned either to aldehydes (MsexOR80 and 33) or to esters (MsexOR8 and 36).

What might be the ecological significance of aldehyde and ester sensing in the life history of *M. sexta*? The aldehydes nonanal and decanal, sensitively detected by MsexOR80 and 33, are components of the floral headspace of at least 20 plant families^45^ and are also emitted from the leaves of plants in the natural habitat of *M. sexta*.^46^ However, aldehydes were neutral stimuli in laboratory wind tunnel experiments,^36^ making it difficult to speculate on the specific importance of these rather ubiquitous volatiles in the ecology of *M. sexta*. On the other hand, esters such as propyl valerate and ethyl hexanoate, sensitively detected by MsexOR8 and 36, are present in the flower headspace of only one (propyl valerate) and four (ethyl hexanoate) plant families, respectively, according to a study that examined nearly 1,000 plant species from 90 families.^45^ Additionally, in the habitat of *M. sexta*, propyl valerate, ethyl hexanoate, ethyl sorbate, and other esters are specifically emitted from the inflorescence of *Agave palmeri*.^46^^,^^47^ These flowers provide abundant nectar with particularly high energy content,^48^ and the majority of the pollen load on the proboscis of wild-caught *M. sexta* in Arizona consists of *A. palmeri* pollen,^49^ indicating that *A. palmeri* is one of the most frequently visited nectar sources by *M. sexta*. In wind tunnel experiments, several esters present in the headspace of *A. palmeri* flowers also induce robust and prolonged feeding behavior.^36^ Although propyl valerate alone was not behaviorally active, it could influence behavior when present in mixtures with other compounds.^10^ Therefore, these rarely occurring esters may be used by *M. sexta* as reliable cues to locate *A. palmeri* flowers, and ORs with particularly high ester sensitivity may have evolved to improve detection of this valuable nectar source.

### Putative glomerular targets of the paralogous ORs in the female antennal lobe

**Figure 3:**
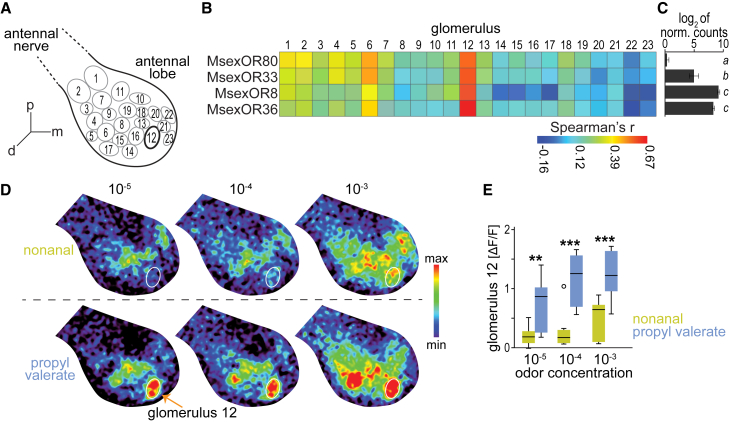
Putative glomerular targets of the paralogous MsexORs in the female antennal lobe (A) Schematic of a right antennal lobe with boundaries of 23 dorsal glomeruli; bold outline marks glomerulus 12 with best corresponding responses to each of the ORs (see B). (B) Heatmap shows correlations (Spearman rank correlation) of odor responses of ectopically expressed MsexORs and 23 olfactory glomeruli upon stimulation with the same 80 odors; imaging results for glomeruli from a study by Bisch-Knaden et al.^36^ See Table S3. (C) Expression of paralogous *MsexORs* in the female antenna. Log_2_ of normalized counts (*n* = 3)^50^; *bars*, mean, *whiskers*, standard deviation; *different letters next to bars*, *p* < 0.001, ANOVA with Tukey-Kramer multiple comparisons test. (D) Representative individual calcium responses to the best ligands of MsexOR80 and 33 (nonanal, upper panel) and MsexOR8 and 36 (propyl valerate, lower panel) at three concentrations; *white outline*, position of glomerulus 12; all images are shown at the same scale. (E) Calcium responses of glomerulus 12 to nonanal (*n* = 7–9) and propyl valerate (*n* = 10–11) at three concentrations; *boxplots*, median, interquartile range and range; *circle*, outlier; ∗∗, *p* < 0.01, ∗∗∗, *p* < 0.001, Mann-Whitney U-test. See Table S4.

Next, we asked whether the OSNs expressing the paralogous *MsexORs* could target any of the 23 previously identified glomeruli in the dorsal part of the female antennal lobe (Figure 3A;^36^). Because the same 80 odorants were tested in both studies, we were able to correlate our current SSR results with these previous calcium imaging results (Figure 3B). Notably, we found that the response of each of the paralogous MsexORs best correlated with the response of a single glomerulus among the 23 glomeruli that could be imaged (glomerulus 12). This finding may indicate that the four *MsexORs* are expressed in a common OSN population projecting to glomerulus 12.

Although in most cases a single odor-selective *OR* is expressed in a single OSN population, there are reports of coexpression of two or more *ORs* in different insects such as flies,^40^^,^^51^ mosquitoes,^52^^,^^53^ and moths.^54^ In flies, several coexpressed *ORs* have been described that are encoded by duplicated genes arranged in a tandem array.^21^^,^^35^ In these cases, the response of the OSN, and thus its target glomerulus, is a combination of the responses of the coexpressed ORs. In previous studies using RNA-seq^29^ and NanoString^50^ techniques, *MsexOR80* expression has not been detected in the tissues examined so far, while the other three paralogous *MsexOR33*, *8*, and *36* are expressed in the antenna (Figure 3C) and may contribute to odor-evoked responses in the antennal lobe. We tested nonanal and propyl valerate, the best ligands of these potentially coexpressed ORs (Figure 2) in calcium imaging experiments (Figure 3D). We found that glomerulus 12 was activated by propyl valerate even at the lowest dose tested (Figure 3E), suggesting that OSN populations innervating glomerulus 12 may indeed express at least one of the ester-sensitive MsexOR8 and 36. However, the activation of glomerulus 12 by nonanal was consistently lower than that by propyl valerate (Figure 3E), suggesting that the aldehyde-sensitive MsexOR33 is either expressed in an OSN population that targets a different glomerulus or that this OR contributes little to the response of glomerulus 12, consistent with the lower antennal expression level of *MsexOR33* compared to *MsexOR8* and *36* (Figure 3C).

### Generation of an *MsexOR36* knock-out line

To further investigate the possible coexpression of the paralogous *MsexORs* and the functional identity of the putative target glomerulus 12, we knocked out one of the receptors and monitored changes in the odor-evoked activation patterns in the antennal lobe. We chose *MsexOR36* because the response spectrum of this OR had the highest correlation with glomerulus 12 (Spearman r = 0.67, Figure 3B), and because it has a diagnostic ligand, β-ocimene, to which none of the other paralogs responded (Figure 1D). A loss of response to β-ocimene in *MsexOR36* knock-out moths and an unaltered response to other odors would therefore reveal the identity of the glomerulus targeted by OSNs expressing this gene, while confirming the coexpression of *MsexOR36* and at least one other olfactory receptor with a similar ligand-binding profile. We performed CRISPR-Cas9-mediated mutagenesis and generated a *MsexOR36* mutant line with an 8 bp deletion, removing the last bp of intron 3 and the first 7 bp of exon 4 (Figure S3A), disrupting an intron-exon junction. This resulted in exon skipping during splicing of the mutant *MsexOR36* gene, similar to a previous report of locust *OR* mutagenesis.^55^ Reverse transcription and sequencing revealed a 105 bp deletion corresponding to exon 4 in the knock-out mRNA (Figures S3B and S3C), which corresponds to a deletion of 9% of the translated protein (35 of 407 amino acids). We predicted the transmembrane domains of MsexOR36 based on the alignment with the structurally characterized OR5 from the bristletail *M. hrabei*^56^ and found that the deletion in mutant *MsexOR36* corresponds to a significant portion of the predicted sixth transmembrane helix (S6) (Figure S3D). S6 is essential for the structural stability of the OR fold and a key component in the formation of the ligand-binding pocket,^56^^,^^57^ making this *MsexOR36* mutant unlikely to be functional.

### Knocking out *MsexOR36* abolishes the response of glomerulus 12 to β-ocimene

**Figure 4:**
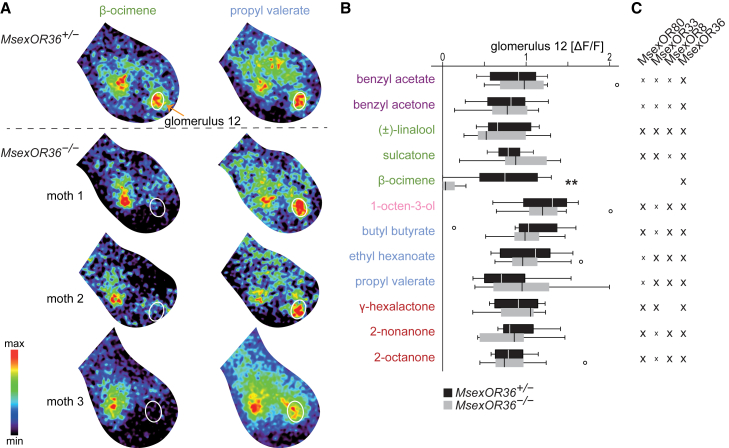
Knocking out *MsexOR36* abolishes the response of glomerulus 12 to β-ocimene (A) Representative calcium responses of one heterozygous and three homozygous mutant moths to the MsexOR36 ligands β-ocimene (left panel) and propyl valerate (right panel); position of glomerulus 12 is marked with a white outline; images from the same individual are displayed at the same intensity scale. (B) Calcium responses of glomerulus 12 in the antennal lobe of *MsexOR36*^+/−^ (*n* = 8) and *MsexOr36*^−/−^ (*n* = 10) moths. *Boxplots*, median, interquartile range and range; *circles*, outliers; odor concentration was 10^−3^; only responses to β-ocimene differed between *MsexOR36*^+/−^ and *MsexOr36*^−/−^ moths; ∗∗, *p* = 0.0062; responses to all other odors: *p* ≥ 0.3, Mann-Whitney U-test. For EAG results, see Figure S4. (C) Responses of MsexOR80, 33, 8 and 36 to best ligands of MsexOR36 in SSR. *X*, ≥ half maximum median response of the OR; *x*, < half maximum response (see Figure 1D); *MsexOR80* expression was not detected in the antenna (Figure 3C), making a contribution of MsexOR80 responses to antennal lobe activation patterns unlikely. See Table S5.

We performed calcium imaging experiments to measure odor-evoked activation in the antennal lobe of heterozygous (*MsexOR36*^+/−^) and homozygous (*MsexOR36*^−/−^) knock-out females upon stimulation with the best ligands of MsexOR36 (12 odors in total). We found that the β-ocimene response of glomerulus 12 seen in *MsexOR36*^+/−^ moths was abolished in *MsexOR36*^−/−^ moths, suggesting that *MsexOR36*-expressing OSNs do indeed target glomerulus 12 (Figure 4A). Other glomeruli were still activated by β-ocimene, as this odorant activates more than one glomerulus^36^ and is detected by more than one OSN population.^58^ Redundant detection of individual odorants was also evident in electroantennogram (EAG) recordings, which represent the pooled response of all OSN populations of the antenna, as these results were similar for both genotypes (Figure S4).

In contrast to β-ocimene, the remaining best ligands of MsexOR36 induced activation in glomerulus 12 of *MsexOR36*^−/−^ moths similar to that of the control genotype (Figure 4B). Because all ligands except β-ocimene were detected by other ORs in the clade (Figure 4C), our calcium imaging results suggest not only that *MsexOR36*-expressing OSNs project to glomerulus 12, but also that *MsexOR36* is coexpressed with at least one of its paralogs in a common OSN population. Although we cannot exclude the possibility that other ORs with a similar receptive range are coexpressed with *MsexOR36*, such functional overlap of a broad ligand binding spectrum seems unlikely as it has not been found in other insects.^2^^,^^4^^,^^38^ Based on the high ester sensitivity and low aldehyde sensitivity of glomerulus 12 (Figure 3E), we speculated before that only the two ester-sensitive paralogs MsexOR8 and 36 are coexpressed and that the aldehyde-sensitive MsexOR33 might be expressed in a different OSN population. However, glomerulus 12 of moths with both MsexOR36^−/−^ and MsexOR36^+/−^ genotypes responded similarly to an odorant (γ-hexalactone, Figure 4C) that was one of the best ligands of MsexOR36 and 33 but was not detected by MsexOR8, therefore suggesting coexpression of all three paralogs.

As an alternative to the coexpression hypothesis, the paralogous ORs could be expressed in distinct OSN populations that either co-converge at glomerulus 12,^59^ or target distinct but densely packed glomeruli^60^ that are indistinguishable by our calcium imaging technique. A direct test of the coexpression hypothesis would require double-labeling RNA fluorescence *in situ* hybridization (RNA FISH) experiments, but because nucleotide identities above 80% lead to cross-reactivity of RNA FISH probes,^21^ the level of sequence similarity between the paralogous ORs (87%–89% nucleotide identity) was too high to perform these tests. However, given the increasing number of reports of coexpression of clustered and duplicated ORs,^21^^,^^52^^,^^53^^,^^59^ all of our results taken together support the idea that *MsexOR36* may be coexpressed with its paralogs.

### Evolutionary history of the expanded *MsexOR* clade

**Figure 5:**
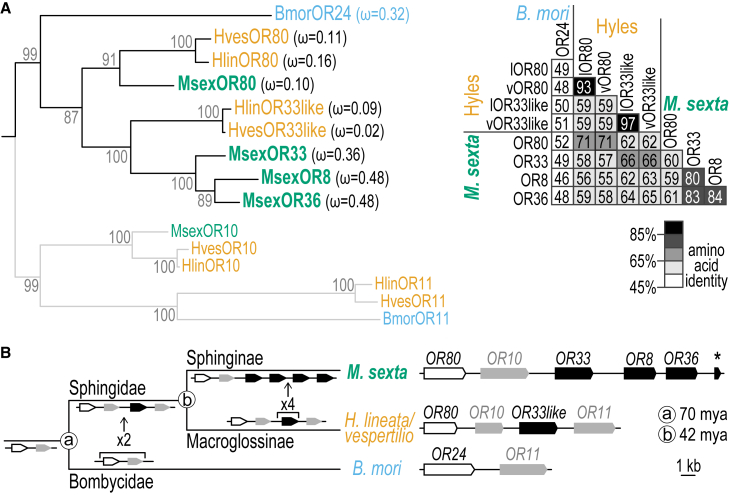
Evolutionary history of the expanded *MsexOR* clade (A) *Left*, maximum likelihood tree of the focal *OR* clade (black branches) and its sister clade (gray branches). Numbers show branch support values from 1,000 bootstrap replicates; ω estimates in brackets next to focal *ORs* indicate the ratio of non-synonymous to synonymous substitutions (ω = d*N*/d*S*). *Right*, matrix of pairwise amino acid identities of focal *ORs* based on MAFFT alignment generated for the phylogenetic tree. Lighter to darker gray values represent lower to higher sequence identities; l, *H. lineata*, v, *H. vespertilio*. (B) Reconstructed gene birth-death dynamics mapped onto the species phylogeny (left, not to scale) and the respective tandem arrays (right). Divergence times of the moth families Sphingidae and Bombycidae (a) and the sphingid subfamilies Sphinginae and Macroglossinae (b) are shown^64^; *H. lineata* and *H. vespertilio* tandem arrays are identical; *asterisk*, pseudogene *MsexOR85*.

The expansion of *M. sexta* ORs studied here was described in a phylogenetic tree that included seven moth and butterfly species.^29^ The closest relative of *M. sexta* in this previous analysis was the silkmoth *B. mori*, which belongs to a different family, making it difficult to reconstruct the evolutionary history of the expanded *M. sexta* OR clade. We therefore annotated orthologs of the clade in two recently published hawkmoth genomes, the white-lined sphinx moth *Hyles lineata*^61^ and the bat hawkmoth *H. vespertilio*^62^ using a semi-automated annotation pipeline.^63^ Phylogenetic reconstruction of orthologous ORs in the three hawkmoth species and in *B. mori* revealed that the Hyles species have an ortholog of MsexOR80 and a single ortholog of the MsexOR33/8/36 group (Figure 5A). This result suggests that OR80 and an ortholog of the OR33/8/36 group were already present in the common ancestor of Hyles and Manduca and that the OR33/8/36 group expanded only after the divergence of the Hyles and Manduca lineages (Figure 5B).

The genomic arrangement of *ORs* belonging to the focal clade (including the studied *BmorOR* and *MsexORs*, Figure 5A, black branches) and its sister clade (Figure 5A, gray branches) allows further inference of gene birth-death dynamics. In each species, all genes of the focal clade and sister clade are located in a single tandem array (Figure 5B). The tandem array consists of only two *ORs* in *B. mori* but is extended in hawkmoths with four genes in the Hyles species and six genes in *M. sexta*. While the *B. mori* tandem array consists of one focal clade gene (*OR24*) and one sister clade gene (*OR11*), the Hyles array carries two focal clade homologs (*OR80* and *OR33like*) interleaved with two sister clade homologs (*OR10* and *OR11*). This suggests that an ancestral tandem array of two *ORs* conserved in *B. mori* has duplicated to an array of four *ORs*. *M. sexta* harbors only a single OR from the sister clade (*OR10)* suggesting that *OR11* was lost after the split of the Hyles and Manduca lineages. In addition to *OR80*, three functional and one pseudogenized member of the focal clade are present in the *M. sexta* tandem array, indicating multiple duplication events in this lineage that gave rise to the present Manduca-specific expansion (Figure 5B). This expansion most likely originated from a Hyles*-OR33like* ancestor, as suggested by the phylogenetic relationships (Figure 5A). We performed selection analyses based on ω (or d*N*/d*S*) estimates to reconstruct the evolutionary dynamics of this group of ORs, and to assess the potential for variation in selective pressure among recent paralogs to confound phylogenetic inference of their relationships. None of the branches of the focal clade showed signatures of positive selection (*p* = 1, aBSREL^65^). However, the genes of the MsexOR33/8/36 group had the highest ω estimates in the focal clade of ORs (Figure 5A). Elevated ω values are a common signature of recently duplicated genes and may be a result of relaxed purifying selection.^66^ To confirm such relaxation, we estimated the selection intensity parameter *k* of the MsexOR33/8/36 group against the Hlin/HvesOR33-like background using RELAX.^67^ We found that the MsexOR33/8/36 group has a reduced selection intensity parameter (k = 0.05; *p* < 0.0001, likelihood-ratio test: 35.54), indicating relaxation of purifying selective strength. This supports the notion that MsexOR33/8/36 represents a recent group of paralogs that arose by gene duplication after the split from the most recent common ancestor shared with Hyles.

Our analyses suggest the following evolutionary history within the focal clade of ORs: *B. mori* has a single ortholog BmorOR24, which probably represents the ancestral state of the clade. After the silkmoth and hawkmoth lineages split 70 million years ago, the ancestral OR duplicated, resulting in two paralogous copies in the clade. After the hawkmoth subfamilies Sphinginae (Manduca) and Macroglossinae (Hyles) diverged 42 million years ago, further duplication events gave rise to the clade of three paralogous ORs in *M. sexta*, i.e., MsexOR33, 8, and 36.

Taken together, the paralogous *M. sexta* ORs studied here encode broadly tuned receptors that fall into two functional groups at lower stimulus doses, one sensitive to aldehydes (MsexOR80 and 33) and the other sensitive to esters (MsexOR8 and 36). The silkmoth ortholog BmorOR24 also responded to both aldehydes and esters (among odors from other chemical classes). However, the four hawkmoth homologs together detected twice as many odorants at higher stimulus doses than BmorOR24, suggesting that the duplicated *M. sexta* genes have undergone both subfunctionalization and neofunctionalization. Based on functional similarities with previous glomerular imaging results, the *MsexORs* (probably with the exception of *MsexOR80*, whose expression could not be detected in the antenna) appear to be coexpressed in an OSN population projecting to the same olfactory glomerulus in the antennal lobe. This hypothesis was supported by calcium imaging data obtained from *MsexOR36* mutant moths. Interestingly, this single target glomerulus (glomerulus 12) was previously identified as one of four glomeruli whose activation levels were positively correlated with the duration of odor-evoked feeding behavior in female *M. sexta*, and esters were one of two chemical classes that induced the strongest feeding behavior in wind tunnel experiments.^36^ Flowers of *A. palmeri*, one of the most valuable and visited nectar sources for hawkmoths in Arizona, attract both *M. sexta* and *H. lineata*. However, while agave pollen accounted for 46% and 70% of the pollen load on the proboscis of *M. sexta* in two consecutive seasons, its contribution to the pollen load on the proboscis of *H. lineata* was only 6% in both years.^49^ These data suggest that *H. lineata* is a more generalist forager than *M. sexta* and is less dependent on finding the ester-rich floral bouquet of *A. palmeri*. Thus, the evolution of the ester-sensitive MsexOR8 and 36, which most likely occurred after the split of the Manduca and Hyles lineages, seems consistent with the hypothesis that the OR gene expansion studied allows for enhanced detection of food cues that are particularly important in the ecology of *M. sexta*.

### Limitations of the study

Our results suggest that the studied clade of *MsexORs* is coexpressed in one population of OSNs. This assumption is supported by results in vinegar flies and mosquitoes showing that coexpressed ORs often belong to gene expansions and are arranged in tandem.^21^^,^^51^^,^^52^^,^^53^^,^^59^ In these studies, coexpression was confirmed by RNA FISH, single-nucleus RNA sequencing, and gene editing methods. Although FISH experiments were not feasible in our case due to the high sequence similarity of the paralogs,^21^ single-nucleus RNA sequencing would be a suitable method to demonstrate coexpression. Furthermore, the generation of transgenic moths with GFP-labeled ORs would allow visualization of the projections of OSNs expressing the paralogous *MsexORs* in the antennal lobe, providing direct evidence for the identity of the targeted glomerulus.

## Resource availability

### Lead contact

Further information and requests for resources and reagents should be directed to and will be fulfilled by the lead contact, Sonja Bisch-Knaden (sbisch-knaden@ice.mpg.de).

### Materials availability

This study did not generate new unique reagents.

### Data and code availability

• All data reported in this paper can be found in Document S1 (Tables S3–S5) and Tables S6 and S7.
• All original code is available in this paper’s supplemental information (Data S1 ZIP file).
• OR tandem arrays of *Hyles lineata* and H. *vespertilio* were annotated in previously published genome assemblies. The data have been deposited at Dryad; accession numbers are publicly available as of the date of publication.
• Any additional information required to reanalyze the data reported in this paper is available from the lead contact upon request.

## Acknowledgments

We thank Christian Klinner, Sofia Lavista-Llanos and Ewald Grosse-Wilde for creating the empty neuron fly line for *MsexOR36*; Kazushige Touhara for providing the vector containing *BmorOR24*; and Hetan Chang, Finote Gjisman, Bhawana Israni, Ian Keesey, Angela Lehmann, Elisa Schuh, and Vignesh Venkateswaran for help and discussions. This study was supported by 10.13039/501100004189Max Planck Society (all authors except P.B.), the 10.13039/100005156Alexander von Humboldt Foundation (J.Z.), and the 10.13039/100000893Simons Foundation (P.B., #718234).

## Author contributions

S.B.-K., B.S.H., and M.T.T.: study conception and design; P.B.: receptor evolution analyses: M.E.E.H., M.T.T., and S.B.: molecular cloning of moth ORs; M.T.T.: receptor de-orphanization; J.Z., M.T.T., and S.B.: generation of receptor knock-out line; S.B.-K.: calcium imaging; M.T.T. and S.B.-K.: data analysis; M.T.T. and S.B.-K.: writing original draft; all authors revised the manuscript.

## Declaration of interests

The authors declare no competing interests.

## STAR★Methods

### Key resources table

**Table:** 

REAGENT or RESOURCE	SOURCE	IDENTIFIER
**Chemicals, peptides, and recombinant proteins**		

80 odorants, see Table S1 in Supplemental information, Document S1		

**Experimental models: Organisms/strains**		

*w*; *Δhalo*/*CyO*	John Carlson (University of Yale, USA)	N/A
*w*; *Δhalo*/*CyO*; *DmelOr22a-Gal4*	John Carlson (University of Yale, USA)	N/A
*Δhalo*; *DmelOr22a-Gal4*/*UAS-BmorOR24*	this study	N/A
*Δhalo*; *DmelOr22a-Gal4*/*UAS-MsexOR80*	this study	N/A
*Δhalo*; *DmelOr22a-Gal4*/*UAS-MsexOR33*	this study	N/A
*Δhalo*; *DmelOr22a-Gal4*/*UAS-MsexOR8*	this study	N/A
*Δhalo*; *DmelOr22a-Gal4*/*UAS-MsexOR36*	this study	N/A

**Oligonucleotides**		

For primers see Table S2 in Supplemental information, Document S1		
OR tandem arrays of *Hyles lineata* and *H. vespertilio* homologous to the OR33 tandem array of *M. sexta*	this study (annotated in previously published genome assemblies)	https://doi.org/10.5061/dryad.tdz08kq7r

**Software and algorithms**		

Software code to analyze functional calcium imaging data (C. G. Galizia, M. Ditzen)	IDL (L3Harris Geospatial)	Data S1 ZIP file

### Experimental model and study participant details

#### *Drosophila melanogaster*

All fly stocks were reared on standard cornmeal-molasses agar medium in clear polystyrene vials in incubators at 25°C, 70% relative humidity and 12 h:12 h light:dark cycle. The *Δhalo* empty neuron fly lines with deletion of *DmelOR22a/b* genes: *w*; *Δhalo*/*CyO* and the Gal4 parental line: *w*; *Δhalo*/*CyO*; *DmelOr22a-Gal4* were kindly provided by John Carlson (University of Yale, USA). A cross of these two lines generated the empty neuron control flies: *w*; *Δhalo*; *DmelOr22a-Gal4*/*+*. The *w*; *Δhalo*/*CyO* line, the double balancer *yw*; *Cyo*/*Bl*; *TM6B*/*TM2* and the newly created *yw*; +; *UAS-ORX*/*TM3 Sb Ser* lines were used for crossings to generate the UAS parental lines for the five *ORs*: *yw/w*; *Δhalo*/*CyO*; *UAS-ORX*. The parental Gal4 and UAS lines were crossed to generate the test flies: *Δhalo*; *DmelOr22a-Gal4*/*UAS-ORX* which expressed moth *ORs* in ab3A neuron in *Δhalo* background. The test flies were selected under CO_2_ anesthesia and then kept for recovery in food vials for at least 48 h before starting the experiment. All tested flies were 2–6 days old.

#### *Manduca sexta*

Egg collection was done by providing *Datura wrightii* plants to mated *M. sexta* females and caterpillars were reared on artificial diet^29^ in a climate chamber with a 16 h:8 h light:dark cycle at 26°C, and 40% relative humidity. Male and female pupae were kept in separate climate chambers with a 16 h:8 h light:dark cycle, at 25°C, and 60% relative humidity (light cycle) or 70% relative humidity (dark cycle).

### Method details

#### Generation of *UAS-ORX* fly lines

*D. melanogaster* lines with insertion of *UAS-ORX* constructs in the 3^rd^ chromosome were generated using phiC31-based integrase system.^68^ Full-length coding sequences of *MsexORs* were previously cloned from *M. sexta* antennal RNA into pCRII cloning vector in our lab.^29^ pBlueScript vector with *BmorOR24* coding sequence insert was a gift from Kazushige Touhara (University of Tokyo, Japan). The *OR* sequences were then subcloned into the integration vector pUASTattB (GenBank ID: EF362409.1) at the multiple cloning sites downstream to *5xUAS* sequence using restriction digestion and ligation. For this, cloning vectors with *OR* inserts were sequenced using standard M13 primers and analyzed in Geneious Prime version 2019.2 to check orientation of the insert and select suitable pairs of restriction enzymes to bring the complete *OR* sequence in correct orientation into the integration vector. Restriction enzymes were purchased from New England Biolabs Inc. (https://www.neb-online.de/) and NEBcloner (https://nebcloner.neb.com/#!/redigest) online tool was used to determine appropriate reaction buffers for double digestion following the manufacturer’s protocol. For each *OR*, the cloning and integration vectors were digested using the same enzymes and run on agarose gel. The fragments of the *OR* insert and the linearized integration vector were purified using E.Z.N.A. gel extraction kit (Omega Bio-tek) and then ligated using T4 DNA ligase (Invitrogen). Correct orientation and sequence of *ORs* downstream of the *5xUAS* construct in the pUASTattB vector was checked by sequencing using the pUASTinsert primers (Table S2; annealing temperature 55°C). The integration vectors carrying *OR* inserts were then sent out to either BestGene Inc. (USA, https://www.thebestgene.com/) or FlyORF (Switzerland, https://www.flyorf-injection.ch/) for injection into embryos of *D. melanogaster ZH-attP-86Fb* strain (BDSC# 24749), which has the *attP* landing site in the 3^rd^ chromosome. We received balanced stocks with genotype *yw*; +; *UAS-ORX*/*TM3 Sb Ser*.

#### Fly preparation and single sensillum recording

A female fly was immobilized in a truncated 200 μL pipette tip with the antennae and part of the eye protruding from the narrow end and sealed with Erkogum dental wax (Erkodent, Germany) on the wider end. This preparation was stuck on a microscope slide using dental wax with the ventral side of the fly facing up. A glass capillary was heat-pulled (using Narishige PC-10 capillary puller, Narishige, Japan) to create a long thin end. The left antenna of the immobilized fly was stretched out by pressing the thin capillary tip between the second and the third antennal segment to access ab3 sensilla. The antenna was then observed under 50× magnification with an Olympus BX51WI upright light microscope. A pair of tungsten electrodes (TW5-3, Science Products, Germany) in a holder (Syntech, Germany) was used for recording and reference. The reference electrode was inserted in the left eye with a manual manipulator (Narishige, Japan) and grounded. The recording electrode attached to a Syntech Universal AC/DC Probe with 10 x amplification and motorized with a piezo manipulator (PM10, Maerzhaeuser Wetzlar) was inserted into a large basiconic sensillum. The action potentials from the neurons in the sensillum were amplified and digitally converted with Syntech IDAC4 and recorded using the Syntech Autospike32 (v3.7) software. The set-up including the microscope and the electrodes was placed on an IG Breadboard (Newport Corporation) in a stainless-steel Faraday cage to reduce vibrations and electrical noise. A stimulus controller (custom built at Max Planck Institute for Chemical Ecology, Germany) was connected to Syntech IDAC4, such that the stimulus trigger started the spike recording. Odor stimulation was done as described under the following sub-heading. The ab3 sensillum was first tentatively identified by its location on the antenna, its morphology and the presence of spikes of two amplitudes. Then, the diagnostic odors 2-heptanone (ligand for the ab3B neuron) and ethyl-3-hydroxybutyrate (ligand for the ab2B neuron), both diluted 10^−3^ v/v in hexane, were used to confirm the correct sensillum type. Absence of DmelOR22a in the ab3A neuron was tested by the absence of the characteristic strong and long-lasting response of wildtype flies to ethyl hexanoate (10^−3^ in hexane), which was different from responses of moth ORs (Figure S5). For screening of moth ORs, 80 odors (Table S1) were presented in random order at dilution of 10^−2^. For each experiment, responses were recorded from a sensillum as long as spike activity was observed; then the electrode was inserted into a different sensillum of the same fly. A maximum of three ab3 sensilla per fly were used. Each odor was presented only once to a given fly. Stimulations with the solvent were done in the beginning, middle and end of an experiment. For dose-response experiments, six dilutions (10^−7^ to 10^−2^ v/v) were presented in increasing concentration.

#### Odor stimulation for single sensillum recording

For odor stimulations, a circular piece of filter paper (Whatman, d = 1.2 cm) was placed inside a glass Pasteur pipettes and 6 μL of odor dilution or solvent was loaded on the filter paper. After 2 min of evaporation, the wide end of the glass pipette was closed with a 1 mL pipette tip sealed with dental wax (Erkodent, Germany). During the experiment, a continuous stream of air (0.5 L/min) mixed into a stream of humidified air (0.5 L/min) was directed toward the antenna. Upon stimulation via a stimulus controller, the dry airstream was replaced by airflow through the odor or solvent loaded glass Pasteur pipette. The stimulus duration was 500 ms. The interstimulus interval was 40 s (for initial odor screening and EAG) or 1 min (for dose response experiments). Filter papers were replaced every day and after a maximum of 3 stimulations (for initial odor screening and EAG) or 2 stimulations (for dose response experiments).

#### Spike analysis

A and B neurons in the ab3 sensilla were differentiated based on spike amplitude, and spikes from A neurons (larger amplitude) were counted using Autospike32 (v3.9). Spikes were also manually counted to correct errors, e.g., when strong responses caused a reduction in spike amplitude. Responses were calculated by subtracting spike counts during the 500 ms pre-stimulus period from spike counts during the 500 ms stimulus period. For solvent-subtracted responses, the average solvent response for each fly was subtracted from each odor response and this value was doubled to obtain the spikes/s value. Spontaneous activity for each test fly was calculated by doubling the number of spikes during 500 ms before stimulus onset.

#### CRISPR/Cas9-mediated gene editing

*MsexOR36* knock-out was generated based on the method established by.^28^ To find target sites for CRISPR/Cas9 gRNA having 5′-N_20_NGG-3′ motif, we used the CHOPCHOP online tool (chopchop.cbu.uib.no), to which the genome version Msex_1.0^69^ was previously submitted. The *MsexOR36* reference gene with OGS2.0 name Msex2.01521-RB was selected.^29^ The online tool searches both strands of the gene for potential target sites and the *M. sexta* genome for potential off-targets. The top two target sites having efficiency scores 62.32 (target 1 at exon 4) and 49.77 (target 2 at exon 2), and having no off-targets, were selected (Figure S3A, Table S2). The crRNA1 and crRNA2 for these two targets, the tracrRNA and Cas9 nuclease were synthesized by Integrated DNA Technologies (Alt-R CRISPR-Cas9 system, IDT). To generate gRNA mixture, 5 μL of 100 μM tracrRNA was combined with 2.5 μL of 200 μM crRNA1 and 2.5 μL of 200 μM crRNA2 and incubated at 95°C for 5 min. Then, 1 μL of gRNA mixture was combined with 0.5 μL 10 mg/mL Cas9, 1 μL NEB Cas9 Buffer and 7.5 μL 0.05% of Amaranth dye - Acid Red 27 (Sigma-Aldrich), and the mixture was incubated at 37°C for 10 min. The final solution was loaded in quartz microcapillaries (Sutter Instruments Co., Item# QF100-50-10) that were pulled using the laser micropipette puller Model P-2000 (Sutter Instrument Co.) to form a sharp tip. The loaded microcapillaries were then used to inject *M. sexta* eggs within 1 h after oviposition. In detail, freshly laid eggs were collected, washed with distilled water, and attached to a microscope slide with double-sided tape so that the eggs were aligned with their micropyle (anterior) facing down. The slide was placed on the stage of a Zeiss AxioZoom V16 stereomicroscope fitted with a digital micro-manipulator and capillary holder (AuraOptik). A loaded capillary was held at a 45° angle and connected to a Narishige IM 300 microinjector connected to a nitrogen source at 0.62 psi. The capillary tip and the eggs were observed and aligned next to each other with a PlanAPO Z0.5X/0.125 FWD objective lens. To inject the eggs, the microscope stage was moved toward the tip of the capillary with a Sycop3 (Zeiss) stage manipulator until the tip could insert the posterior part of the egg. The injected eggs were kept in the rearing chamber for 2 days and then gently removed from the slide before the caterpillars began to hatch. Ten to 12 days after hatching, the anal horns of the caterpillars were collected with microscissors and each caterpillar was kept in a separate plastic box with food. MyTaq Extract-PCR Kit (Bioline) was used to extract DNA from horn tissue and to perform PCR to amplify the genomic region surrounding each of the target sites (Table S2). The PCR settings were 95°C (1 min), 35 cycles of 95°C (15 s), 51°C (15 s) and 72°C (20 s), and final extension of 72°C (10 min). Five μL of the PCR products were used for making heteroduplexes; followed by T7 endonuclease I (NEB) digestion and gel electrophoresis. Digested products indicate heteroduplexes formed by somatic mosaic mutations. For samples with somatic mutations, the PCR products were cloned into pCR2.1 vector (TOPO cloning kit, Invitrogen) and transformed into OneShot TOP10 competent cells. Eight colonies were picked for each sample and Sanger sequencing was done to identify mutations. Caterpillars with frameshift mutations were reared to adulthood and crossed with wildtype *M. sexta*. The G1 offspring was screened for inherited mutations. We found somatic mosaic mutations at both sites but only target 1 mutations were inherited by G1 offspring. Heterozygous G1 individuals carrying the same type of mutation were crossed to obtain a stable, homozygous knock-out line (*MsexOR36*^*−/−*^). A heterozygous line (*MsexOR36*^+/−^) and a wildtype line kept under identical conditions served as controls in electrophysiological and calcium imaging experiments.

#### RT-PCR

Total RNA was extracted from the antennae of wildtype and *MsexOR36*^*−/−*^ moths using Direct-zol RNA miniprep kit (Zymo Research, Germany). One μg of the purified RNA was reverse transcribed to cDNA using SuperScript III First Strand Synthesis Supermix for qRT-PCR (Invitrogen). The cDNA was then PCR amplified using *MsexOR36* CDS primers spanning from exon 1 to exon 6 (Table S2) using MyTaq HS Red Mix (Bioline). The PCR settings were 95°C (1 min), 35 cycles of 95°C (15 s), 59°C (15 s) and 72°C (45 s), and final extension of 72°C (7 min). The product was run on 1% agarose gel and the bands close to 1 kb were extracted and sequenced using the same *MsexOR36* CDS primers.

#### Preparation for calcium imaging experiments

Female moths were tested on day 3 after eclosion. On day 2, moths were placed in a 15 mL plastic tube with the tip cut open. The head protruded at the narrow end and was fixed with dental wax. Labial palps and proboscis were also fixed with dental wax to reduce movement artifacts during experiments. A window was cut in the head capsule between the compound eyes and tissue covering the brain was removed until the antennal lobes were visible. Fifty μL Pluronic F-127 (Invitrogen) was added to 50 μg of the membrane-permeant form of a fluorescent calcium indicator (Calcium Green-1 AM, Invitrogen) and the solution was sonicated for 10 min. Then, 800 μL physiological saline solution^70^ was added and sonicated again for 10 min. Twenty μL of this dye solution was applied to the exposed brain, and the preparation was incubated in a humid chamber for 45 min at room temperature. Then, we rinsed the brain several times with physiological saline solution to remove excess dye and stored the moths at 4°C overnight to calm them down and reduce their movements. Imaging experiments were performed the following day (day 3 after eclosion).

#### Calcium imaging

The imaging setup consisted of a CCD camera (Olympus U-CMAD3) mounted to an upright microscope (Olympus BX51WI) equipped with a water immersion objective (Olympus, 10x/0.30). Calcium Green-1 AM was excited at 475 nm (500 nm shortpass optical filter; xenon arc lamp, Polychrome V, Till Photonics), and fluorescence was detected at 490/515 nm (dichroic longpass/longpass). The setup was controlled by the software Tillvision v4.6 (Till Photonics). 4-fold symmetrical binning resulted in an image size of 344 x 260 pixels, with one pixel corresponding to an area of 4 μm × 4 μm.

#### Odor stimulation for calcium imaging

To create a functional map of glomeruli in the antennal lobe, we first tested 19 diagnostic odors^36^ in each animal. Then, we tested the 12 best ligands of MsexOR36 (≥ half maximum median response at a dilution of 10^−2^) diluted in mineral oil. The immobilized moth was placed upright under the microscope. A glass tube (d = 5 mm) was directed perpendicular to one antenna and delivered a constant stream of charcoal-filtered, moistened air (0.5 L/min). Two glass pipettes were inserted through small holes in the tube. One pipette (inserted 5.5 cm from end of tube) was empty and added clean air to the continuous airstream (0.5 L/min). This airflow could be automatically switched (Syntech Stimulus Controller CS-55) to the second pipette (inserted 3.5 cm from the end of the tube) containing an odor-laden filter paper. This procedure did not alter the airflow reaching the antenna during odor stimulation, thus reducing mechanical interference. An odor stimulus trial lasted 10 s and was recorded at a sampling rate of 4 Hz, corresponding to 40 frames. The time course of an odor stimulus trial was as follows: 2 s clean airflow (frames 1–8), 2 s odor airflow (frames 9–16), and 6 s clean airflow (frames 17–40). Odors were presented with at least 1 min interstimulus interval to avoid adaptation. The sequence of stimuli varied from animal to animal, and a mineral oil control stimulus was presented at the beginning and end of the sequence.

#### Processing of calcium imaging data

An odor stimulus trial resulted in a series of 40 consecutive frames that were analyzed with a custom-written software (IDL, L3Harris Geospatial, see Data S1 ZIP file).^71^ Several processing steps were applied to improve the signal-to-noise ratio: (1) background correction: background activity was defined as the average fluorescence (F) of frames 3–7 (i.e., before stimulus onset) and was subtracted from the fluorescence of each frame. This background-corrected value (deltaF) was divided by the background fluorescence to obtain the relative changes of fluorescence over background fluorescence for each frame (deltaF/F). (2) Bleaching correction: the fluorescent dye bleached slowly during light exposure, so we subtracted from each frame an exponential decay curve estimated from the bleaching of frames 3–7 and frames 26–40 (i.e., before and after stimulus and response). (3) Median filtering: a spatial median filter with a width of 7 pixels was applied to remove outliers. (4) Movement correction: possible shifts of the antennal lobe from one odor stimulus trial to the next, were corrected by aligning frame 20 of each trial to frame 20 of the median trial in a given animal. The outline of the antennal lobe and the remains of the trachea served as a guide for this movement correction procedure. Increased neuronal activity, indicated by an increase in intracellular calcium concentration after odor stimulation, resulted in spatially restricted spots of increased fluorescence in the antennal lobe. At the center of each activity spot, the average deltaF/F was recorded in an area the size of a small to medium-sized glomerulus (60 μm × 60 μm). Time traces of deltaF/F were averaged over three consecutive frames for each activity spot. In these smoothed time traces, the maximum deltaF/F after stimulus onset was determined. The average of the maximum value and the value before and after the maximum were calculated and defined as the animal’s response to the odor stimulation at the given activity spot.

#### Analysis of activity patterns in the antennal lobe

For each animal, an individual schematic of activity spots was constructed by analyzing the activation patterns evoked by the diagnostic odorants, resulting in 23 spots that could be consistently identfied.^36^ The responses in these 23 putative glomeruli were calculated for all odor stimulus trials in a given animal, and the average responses evoked by the solvent trials were subtracted. In this way, the median net response evoked by an odor in each glomerulus could be calculated across animals.

#### Moth antenna preparation and electroantennography (EAG)

Female moths were tested on day 3 after eclosion. Microscissors were used to cut an antenna at the base and the tip. Both ends of the antenna were inserted into two glass capillaries (outer d = 1.5 mm, inner d = 0.84 mm, World Precision Instruments) heat-pulled with (Narishige PC-10 capillary puller) filled with *M. sexta* physiological saline solution.^70^ A silver wire (Ag-AgCl) attached to an electrode holder was inserted at the other end of the glass capillaries. The electrode at the tip of the antenna was connected to a Syntech Universal AC/DC probe with 10 x gain, and the electrode at the base of the antenna was grounded. The electrical signals from the antenna were digitally converted with Syntech IDAC4 and recorded with Autospike32 (v3.7). The antennal preparation was placed in a Faraday chamber, and a Syntech Stimulus Controller v2.7 CS-55 was used to present odor stimuli (as previously described) and simultaneously record the EAG traces. Odor stimulation was performed as previously described. The 12 best ligands of MsexOR36 were tested in random order at 10^−3^ v/v dilution in hexane. The 10^−3^ dilution was chosen based on pilot experiments to avoid saturation of the EAG responses and thus to be able to detect potential small changes in sensitivity between genotypes. EAG traces were analyzed with Autospike32 (v3.9) by measuring the maximum decrease in voltage after stimulus onset.

#### Reconstruction of the evolutionary history of the *M. sexta* odorant receptor expansion

The OR tandem arrays of two Hyles species were annotated (see key resources table) in previously published genome assemblies^61^^,^^62^ using a semi-automated pipeline developed for the annotation of multi-gene chemosensory gene families.^63^ ORs were aligned with MAFFT^72^ using the L-INS-I algorithm with the -maxiterate option set to 1000.^73^ Maximum Likelihood trees were inferred under a JTT + G substitution model for each family with 10 independent ML searches and 1000 bootstrap replicates. We tested for positive selection at all tips of the gene tree using the adaptive branch-site relative effects-likelihood algorithm as implemented in HYPHY.^74^ We Bonferroni-corrected the resulting *p*-values for multiple testing, and derived ω (d*N*/d*S*) from the distributions over the estimated rate classes and weights. We then tested for relaxed selection of the MsexOR33/8/36 clade by estimating the selection intensity parameter *k* using RELAX^67^ followed by a Likelihood-Ratio Test between the null model and a model of relaxed selection with the *M. sexta* clade as foreground against HylesOR33like as background.

### Quantification and statistical analysis

Sample sizes and statistical tests are indicated in the text and figure legends. Statistical tests were performed using GraphPad InStat v3 and PAST v4. For the NMDS plot and ANOSIM analysis in Figure 1F, individual missing data per odor (156 out of 4240 values) were replaced with the median of the corresponding group. Graphs were generated in IBM SPSS Statistics v25.0, R v4.1.2, Rstudio, PAST v4, and Microsoft Excel. Sequence alignments were performed in Geneious Prime 2019.2. All figures were created in Adobe Illustrator CS5.

## Supplemental information

Document S1. Figures S1–S5 and Tables S1–S5

Table S6. Results of single sensillum recordings from ectopically expressed BmorOR24 and MsexOR80,33,8,36 to 80 odorants, related to Figure 1D

Table S7. Results of dose-response experiments from ectopically expressed MsexOR80,33,8,36 to their best ligands, related to Figure 2

Data S1. Software code to analyze functional calcium imaging data using IDL, related to STAR Methods

## Footnotes

Supplemental information can be found online at https://doi.org/10.1016/j.isci.2024.111317.

## References

[1] Hansson B.S., Stensmyr M.C. (2011). Evolution of insect olfaction. Neuron.

[2] de Fouchier A., Walker W.B., Montagné N., Steiner C., Binyameen M., Schlyter F., Chertemps T., Maria A., François M.C., Monsempes C. (2017). Functional evolution of Lepidoptera olfactory receptors revealed by deorphanization of a moth repertoire. Nat. Commun..

[3] Ebrahim S.A.M., Dweck H.K.M., Stökl J., Hofferberth J.E., Trona F., Weniger K., Rybak J., Seki Y., Stensmyr M.C., Sachse S. (2015). *Drosophila* avoids parasitoids by sensing their semiochemicals *via* a dedicated olfactory circuit. PLoS Biol..

[4] Hallem E.A., Carlson J.R. (2006). Coding of odors by a receptor repertoire. Cell.

[5] Stensmyr M.C., Dweck H.K.M., Farhan A., Ibba I., Strutz A., Mukunda L., Linz J., Grabe V., Steck K., Lavista-Llanos S. (2012). A conserved dedicated olfactory circuit for detecting harmful microbes in *Drosophila*. Cell.

[6] Zhang D.D., Löfstedt C. (2015). Moth pheromone receptors: gene sequences, function, and evolution. Frontiers in Ecology and Evolution.

[7] Sato K., Pellegrino M., Nakagawa T., Nakagawa T., Vosshall L.B., Touhara K. (2008). Insect olfactory receptors are heteromeric ligand-gated ion channels. Nature.

[8] Haverkamp A., Hansson B.S., Knaden M. (2018). Combinatorial codes and labeled lines: how insects use olfactory cues to find and judge food, mates, and oviposition sites in complex environments. Front. Physiol..

[9] Malnic B., Hirono J., Sato T., Buck L.B. (1999). Combinatorial receptor codes for odors. Cell.

[10] Bruce T.J.A., Pickett J.A. (2011). Perception of plant volatile blends by herbivorous insects - finding the right mix. Phytochemistry.

[11] Brand P., Robertson H.M., Lin W., Pothula R., Klingeman W.E., Jurat-Fuentes J.L., Johnson B.R. (2018). The origin of the odorant receptor gene family in insects. Elife.

[12] McKenzie S.K., Kronauer D.J.C. (2018). The genomic architecture and molecular evolution of ant odorant receptors. Genome Res..

[13] Andersson M.N., Löfstedt C., Newcomb R.D. (2015). Insect olfaction and the evolution of receptor tuning. Frontiers in Ecology and Evolution.

[14] Ramdya P., Benton R. (2010). Evolving olfactory systems on the fly. Trends Genet..

[15] Robertson H.M., Douglas A.E. (2019).

[16] Bastide H., Legout H., Dogbo N., Ogereau D., Prediger C., Carcaud J., Filée J., Garnery L., Gilbert C., Marion-Poll F. (2024). The genome of the blind bee louse fly reveals deep convergences with its social host and illuminates *Drosophila* origins. Curr. Biol..

[17] Gardiner A., Barker D., Butlin R.K., Jordan W.C., Ritchie M.G. (2008). *Drosophila* chemoreceptor gene evolution:: selection, specialization and genome size. Mol. Ecol..

[18] McBride C.S., Arguello J.R., O'Meara B.C. (2007). Five drosophila genomes reveal nonneutral evolution and the signature of host specialization in the chemoreceptor superfamily. Genetics.

[19] Kirkness E.F., Haas B.J., Sun W., Braig H.R., Perotti M.A., Clark J.M., Lee S.H., Robertson H.M., Kennedy R.C., Elhaik E. (2010). Genome sequences of the human body louse and its primary endosymbiont provide insights into the permanent parasitic lifestyle. Proc. Natl. Acad. Sci. USA.

[20] Jongepier E., Séguret A., Labutin A., Feldmeyer B., Gstöttl C., Foitzik S., Heinze J., Bornberg-Bauer E. (2022). Convergent loss of chemoreceptors across independent origins of slave-making in ants. Mol. Biol. Evol..

[21] Auer T.O., Álvarez-Ocaña R., Cruchet S., Benton R., Arguello J.R. (2022). Copy number changes in co-expressed odorant receptor genes enable selection for sensory differences in drosophilid species. Nat. Ecol. Evol..

[22] Hou X.Q., Yuvaraj J.K., Roberts R.E., Zhang D.D., Unelius C.R., Löfstedt C., Andersson M.N. (2021). Functional evolution of a bark beetle odorant receptor clade detecting monoterpenoids of different ecological origins. Mol. Biol. Evol..

[23] Matsunaga T., Reisenman C.E., Goldman-Huertas B., Brand P., Miao K., Suzuki H.C., Verster K.I., Ramírez S.R., Whiteman N.K. (2022). Evolution of olfactory receptors tuned to mustard oils in herbivorous Drosophilidae. Mol. Biol. Evol..

[24] Engsontia P., Sangket U., Chotigeat W., Satasook C. (2014). Molecular evolution of the odorant and gustatory receptor genes in Lepidopteran insects: implications for their adaptation and speciation. J. Mol. Evol..

[25] Lynch M., Conery J.S. (2000). The evolutionary fate and consequences of duplicate genes. Science.

[26] Rastogi S., Liberles D.A. (2005). Subfunctionalization of duplicated genes as a transition state to neofunctionalization. BMC Evol. Biol..

[27] He X., Zhang J. (2005). Rapid subfunctionalization accompanied by prolonged and substantial neofunctionalization in duplicate gene evolution. Genetics.

[28] Fandino R.A., Haverkamp A., Bisch-Knaden S., Zhang J., Bucks S., Nguyen T.A.T., Schröder K., Werckenthin A., Rybak J., Stengl M. (2019). Mutagenesis of odorant coreceptor Orco fully disrupts foraging but not oviposition behaviors in the hawkmoth *Manduca sexta*. Proc. Natl. Acad. Sci. USA.

[29] Koenig C., Hirsh A., Bucks S., Klinner C., Vogel H., Shukla A., Mansfield J.H., Morton B., Hansson B.S., Grosse-Wilde E. (2015). A reference gene set for chemosensory receptor genes of *Manduca sexta*. Insect Biochem. Mol. Biol..

[30] Guo M., Du L., Chen Q., Feng Y., Zhang J., Zhang X., Tian K., Cao S., Huang T., Jacquin-Joly E. (2021). Odorant receptors for detecting flowering plant cues are functionally conserved across moths and butterflies. Mol. Biol. Evol..

[31] Wicher D., Morinaga S., Halty-deLeon L., Funk N., Hansson B., Touhara K., Stengl M. (2017). Identification and characterization of the bombykal receptor in the hawkmoth *Manduca sexta*. J. Exp. Biol..

[32] Zhang J., Komail Raza S.A., Wei Z., Keesey I.W., Parker A.L., Feistel F., Chen J., Cassau S., Fandino R.A., Grosse-Wilde E. (2022). Competing beetles attract egg laying in a hawkmoth. Curr. Biol..

[33] Hou X.Q., Jia Z., Zhang D.D., Wang G. (2024). Odorant receptor orthologues from moths display conserved responses to *cis*-jasmone. Insect Sci..

[34] Tanaka K., Uda Y., Ono Y., Nakagawa T., Suwa M., Yamaoka R., Touhara K. (2009). Highly selective tuning of a silkworm olfactory receptor to a key mulberry leaf volatile. Curr. Biol..

[35] Auer T.O., Khallaf M.A., Silbering A.F., Zappia G., Ellis K., Álvarez-Ocaña R., Arguello J.R., Hansson B.S., Jefferis G.S.X.E., Caron S.J.C. (2020). Olfactory receptor and circuit evolution promote host specialization. Nature.

[36] Bisch-Knaden S., Dahake A., Sachse S., Knaden M., Hansson B.S. (2018). Spatial representation of feeding and oviposition odors in the brain of a hawkmoth. Cell Rep..

[37] Dobritsa A.A., van der Goes van Naters W., Warr C.G., Steinbrecht R.A., Carlson J.R. (2003). Integrating the molecular and cellular basis of odor coding in the *Drosophila* antenna. Neuron.

[38] Carey A.F., Wang G., Su C.Y., Zwiebel L.J., Carlson J.R. (2010). Odorant reception in the malaria mosquito *Anopheles gambiae*. Nature.

[39] Chang H., Unni A.P., Tom M.T., Cao Q., Liu Y., Wang G., Llorca L.C., Brase S., Bucks S., Weniger K. (2023). Odorant detection in a locust exhibits unusually low redundancy. Curr. Biol..

[40] Couto A., Alenius M., Dickson B.J. (2005). Molecular, anatomical, and functional organization of the *Drosophila* olfactory system. Curr. Biol..

[41] Fishilevich E., Vosshall L.B. (2005). Genetic and functional subdivision of the *Drosophila* antennal lobe. Curr. Biol..

[42] Bhandawat V., Olsen S.R., Gouwens N.W., Schlief M.L., Wilson R.I. (2007). Sensory processing in the *Drosophila* antennal lobe increases reliability and separability of ensemble odor representations. Nat. Neurosci..

[43] Hallem E.A., Ho M.G., Carlson J.R. (2004). The molecular basis of odor coding in the *drosophila* antenna. Cell.

[44] Geithe C., Noe F., Kreissl J., Krautwurst D. (2017). The broadly tuned odorant receptor OR1A1 is highly selective for 3-methyl-2,4-nonanedione, a key food odorant in aged wines, tea, and other foods. Chem. Senses.

[45] Knudsen J.T., Eriksson R., Gershenzon J., Ståhl B. (2006). Diversity and distribution of floral scent. Bot. Rev..

[46] Bisch-Knaden S., Rafter M.A., Knaden M., Hansson B.S. (2022). Unique neural coding of crucial versus irrelevant plant odors in a hawkmoth. Elife.

[47] Raguso R.A. (2004). Why are some floral nectars scented?. Ecology.

[48] Riffell J.A., Alarcón R., Abrell L., Davidowitz G., Bronstein J.L., Hildebrand J.G. (2008). Behavioral consequences of innate preferences and olfactory learning in hawkmoth-flower interactions. Proc. Natl. Acad. Sci. USA.

[49] Alarcon R., Davidowitz G., Bronstein J.L. (2008). Nectar usage in a southern Arizona hawkmoth community. Ecol. Entomol..

[50] Tom M.T., Cortés Llorca L., Bucks S., Bisch-Knaden S., Hansson B.S. (2022). Sex- and tissue-specific expression of chemosensory receptor genes in a hawkmoth. Frontiers in Ecology and Evolution.

[51] Goldman A.L., Van der Goes van Naters W., Lessing D., Warr C.G., Carlson J.R. (2005). Coexpression of two functional odor receptors in one neuron. Neuron.

[52] Karner T., Kellner I., Schultze A., Breer H., Krieger J. (2015). Co-expression of six tightly clustered odorant receptor genes in the antenna of the malaria mosquito *Anopheles gambiae*. Front. Ecol. Evol..

[53] Adavi E.D., dos Anjos V.L., Kotb S., Metz H.C., Tian D., Zhao Z., Zung J.L., Rose N.H., McBride C.S. (2024). Olfactory receptor coexpression and co-option in the dengue mosquito. bioRxiv.

[54] Anderson A.R., Wanner K.W., Trowell S.C., Warr C.G., Jaquin-Joly E., Zagatti P., Robertson H., Newcomb R.D. (2009). Molecular basis of female-specific odorant responses in *Bombyx mori*. Insect Biochem. Mol. Biol..

[55] Chen D., Tang J.X., Li B., Hou L., Wang X., Kang L. (2018). CRISPR/Cas9-mediated genome editing induces exon skipping by complete or stochastic altering splicing in the migratory locust. BMC Biotechnol..

[56] del Mármol J., Yedlin M.A., Ruta V. (2021). The structural basis of odorant recognition in insect olfactory receptors. Nature.

[57] Zhao J., Chen A.Q., Ryu J., del Mármol J. (2024). Structural basis of odor sensing by insect heteromeric odorant receptors. Science.

[58] Ghaninia M., Olsson S.B., Hansson B.S. (2014). Physiological organization and topographic mapping of the antennal olfactory sensory neurons in female hawkmoths, *Manduca sexta*. Chem. Senses.

[59] Herre M., Goldman O.V., Lu T.C., Caballero-Vidal G., Qi Y., Gilbert Z.N., Gong Z., Morita T., Rahiel S., Ghaninia M. (2022). Non-canonical odor coding in the mosquito. Cell.

[60] Prieto-Godino L.L., Rytz R., Cruchet S., Bargeton B., Abuin L., Silbering A.F., Ruta V., Dal Peraro M., Benton R. (2017). Evolution of acid-sensing olfactory circuits in drosophilids. Neuron.

[61] Godfrey R.K., Britton S.E., Mishra S., Goldberg J.K., Kawahara A.Y. (2023). A high-quality, long-read genome assembly of the whitelined sphinx moth (Lepidoptera: Sphingidae: *Hyles lineata*) shows highly conserved melanin synthesis pathway genes. G3 (Bethesda)..

[62] Pippel M., Jebb D., Patzold F., Winkler S., Vogel H., Myers G., Hiller M., Hundsdoerfer A.K. (2020). A highly contiguous genome assembly of the bat hawkmoth *Hyles vespertilio* (Lepidoptera: Sphingidae). GigaScience.

[63] Brand P., Ramírez S.R. (2017). The evolutionary dynamics of the odorant receptor gene family in corbiculate bees. Genome Biol. Evol..

[64] Kawahara A.Y., Plotkin D., Espeland M., Meusemann K., Toussaint E.F.A., Donath A., Gimnich F., Frandsen P.B., Zwick A., dos Reis M. (2019). Phylogenomics reveals the evolutionary timing and pattern of butterflies and moths. Proc. Natl. Acad. Sci. USA.

[65] Smith M.D., Wertheim J.O., Weaver S., Murrell B., Scheffler K., Kosakovsky Pond S.L. (2015). Less is more: an adaptive branch-site random effects model for efficient detection of episodic diversifying selection. Mol. Biol. Evol..

[66] Kondrashov F.A., Rogozin I.B., Wolf Y.I., Koonin E.V. (2002). Selection in the evolution of gene duplications. Genome Biol..

[67] Wertheim J.O., Murrell B., Smith M.D., Kosakovsky Pond S.L., Scheffler K. (2015). RELAX: detecting relaxed selection in a phylogenetic framework. Mol. Biol. Evol..

[68] Bischof J., Maeda R.K., Hediger M., Karch F., Basler K. (2007). An optimized transgenesis system for *Drosophila* using germ-line-specific phiC31 integrases. Proc. Natl. Acad. Sci. USA.

[69] Kanost M.R., Arrese E.L., Cao X., Chen Y.R., Chellapilla S., Goldsmith M.R., Grosse-Wilde E., Heckel D.G., Herndon N., Jiang H. (2016). Multifaceted biological insights from a draft genome sequence of the tobacco hornworm moth, *Manduca sexta*. Insect Biochem. Mol. Biol..

[70] Christensen T.A., Hildebrand J.G. (1987). Male-specific, sex pheromone-selective projection neurons in the antennal lobes of the moth *Manduca sexta*. J. Comp. Physiol..

[71] Galizia C.G., Vetter R.S., Christensen T.A. (2005). Methods in insect sensory neuroscience.

[72] Katoh K., Misawa K., Kuma K.i., Miyata T. (2002). MAFFT: a novel method for rapid multiple sequence alignment based on fast Fourier transform. Nucleic Acids Res..

[73] Katoh K., Kuma K.i., Toh H., Miyata T. (2005). MAFFT version 5: improvement in accuracy of multiple sequence alignment. Nucleic Acids Res..

[74] Pond S.L.K., Frost S.D.W., Muse S.V. (2005). HyPhy: hypothesis testing using phylogenies. Bioinformatics.

