# Supplementary material for: Gene expansion in the hawkmoth Manduca sexta drives evolution of food-associated odorant receptors
Source: iScience. 2024 Nov 4;27(12):111317. doi: 10.1016/j.isci.2024.111317 (PMC11617253; doi:10.1016/j.isci.2024.111317)
Supplement: Document S1. Figures S1–S5 and Tables S1–S5 [file mmc1.pdf]

iScience, Volume *27*

Supplemental information

Gene expansion in the hawkmoth *Manduca sexta* drives evolution of food-associated odorant receptors

Megha Treesa Tom, Philipp Brand, Sascha Bucks, Jin Zhang, Mario Ernesto Escobar Huezo, Bill S. Hansson, and Sonja Bisch-Knaden

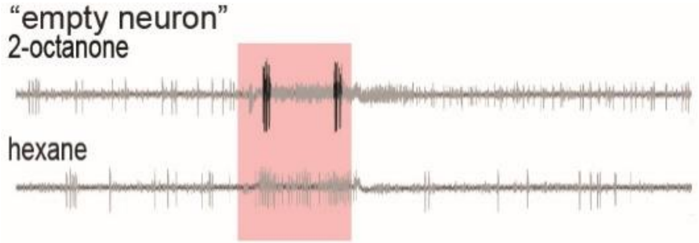

Figure S1. Representative SSR data from the empty ab3 A neuron. (Related to Figure 1B)**.** A burst response to 2-octanone together with the corresponding solvent (hexane) response from the same fly is shown. *Large black spikes*, A neuron; *small gray spikes*, B neuron; *red background*, stimulus duration (500 ms).

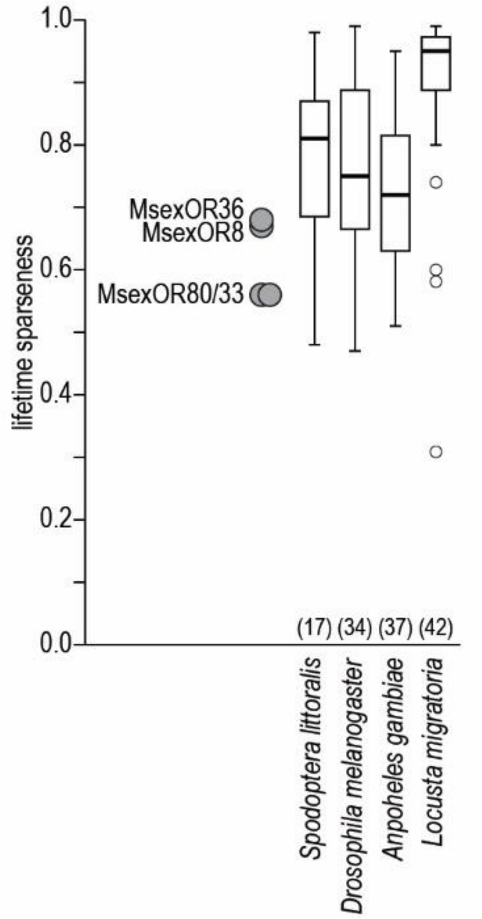

Figure S2. Comparison of lifetime sparseness values of the four de-orphanized MsexORs with data from other insect species. (Related to Figure 1E). *Circles filled in grey,* lifetime sparseness of MsexOR80, 33, 8, and 36 (data from Fig. 1D); *boxplots*, median, interquartile range and range of values of ORs from different insect species; *empty circles*, outliers; *numbers in brackets*, number of ORs from each species. Values were either taken from or calculated using data from [1-4]. Thirteen ORs of *A. gambiae* which gave inhibitory responses to majority of tested odorants [1] were excluded for this analysis.

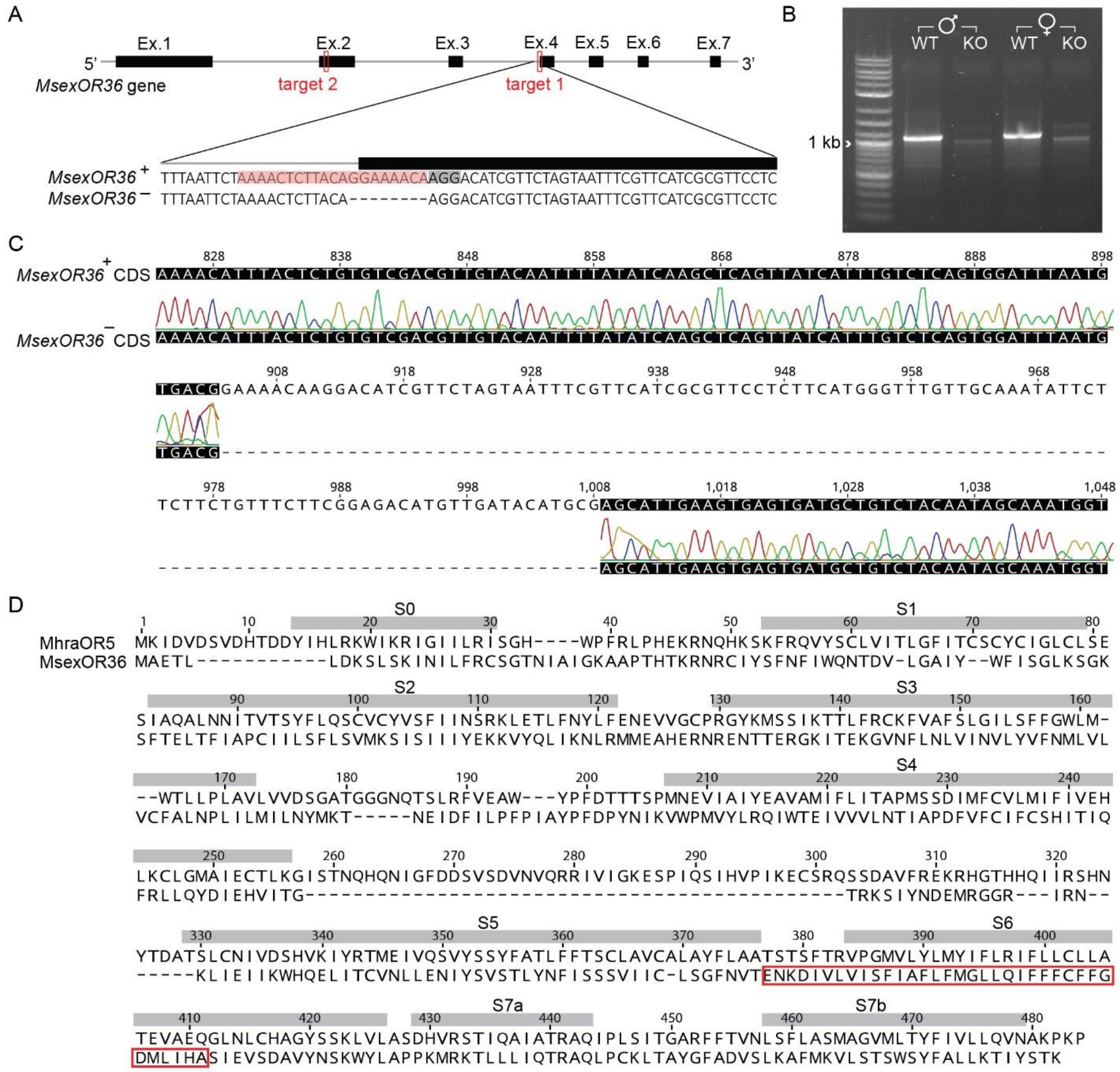

Figure S3. Mutagenesis of *MsexOR36*. A. (Related to Figure 4). *MsexOR36* gene with black boxes depicting exons 1-7 and red boxes showing the two target sites (see Materials and methods) for CRISPR/Cas9 technique. A knock-out line (*MsexOR36*$^{-/-}$) with a mutation at the target site 1 was established. In *MsexOR36*$^{+}$ (wildtype allele) sequence target 1 is highlighted with red and the 3' protospacer adjacent motif (PAM) sequence AGG in grey. *MsexOR36*$^{-}$ (knock-out allele) shows an 8 bp deletion disrupting the intron 3-exon 4 junction. **B.** Gel-electrophoresis image showing RT-PCR products of *MsexOR36* from antennae. The first well has 1 kb plus ladder (NEB) followed by *MsexOR36*$^{+/+}$ (WT; 1115 bp expected) and *MsexOR36*$^{-/-}$ (KO) moth samples. The knock-out samples lack bands at the corresponding position of the wildtype samples. Shift in knock-out band on a 1% agarose gel suggested a deletion larger than seven bases from the mRNA. **C.** Sequencing result for RT-PCR product from *MsexOR36*$^{-/-}$ mapped to reference coding sequence (LN885127.1, *MsexOR36*$^{+}$). Nucleotides 824-1048 of reference are shown. Black highlight denotes identical nucleotides, '-' denotes gap in sequence and the chromatogram shows the intensity of nucleotide signals. **D.** Protein alignment of bristletail receptor MhraOR5 and MsexOR36. Grey bars are helices S0-S7, based on the structure of MhraOR5 [5]. S1-S7a/b are the seven transmembrane helices. Red boxes mark the 35 amino acids which were deleted in the *MsexOR36* knock-out. Figures C and D were created in Geneious Prime version 2019.2.

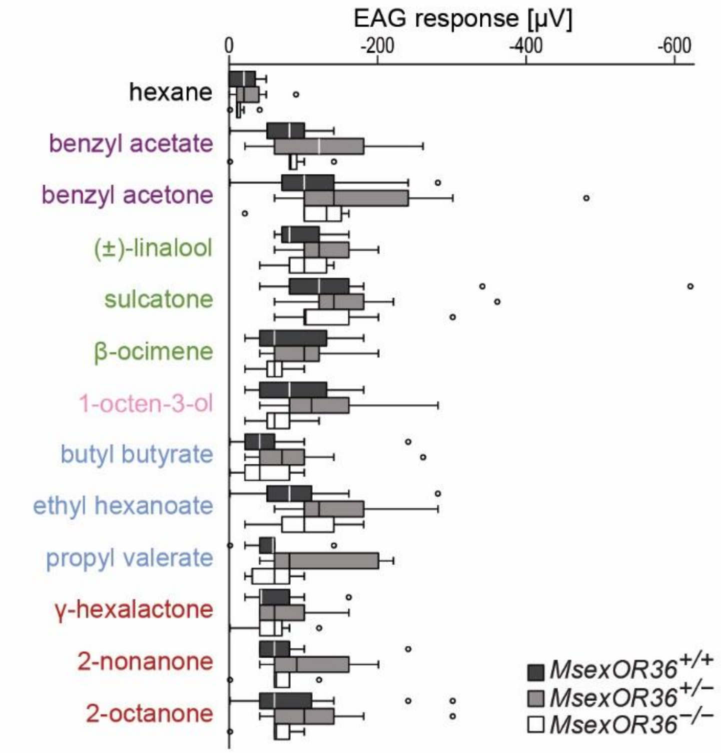

Figure S4. Knocking-out *MsexOR36* does not alter odor responses of female antennae. (Related to Figure 4). EAG responses of moths to the solvent hexane and the best ligands of MsexOR36; *boxplots*, median, interquartile range and range; *circles*, outliers. No difference was found between the three genotypes ($p>0.05$, Kruskal-Wallis test); n=9 (*MsexOR36*$^{+/+}$), n=10 (*MsexOR36*$^{+/-}$) and n=8 (*MsexOR36*$^{-/-}$).

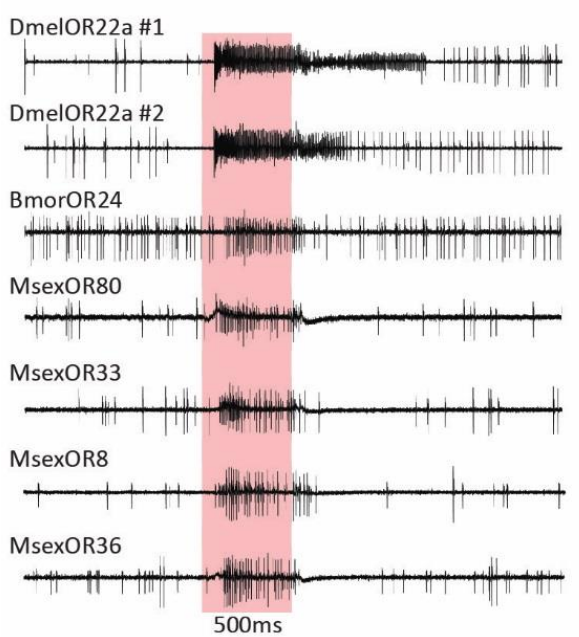

Figure S5. Representative SSR data showing responses of ab3 sensilla to ethyl hexanoate. (Related to Method details: Fly preparation and single sensillum recording). Red background denotes stimulus duration. In each recording, spikes of two amplitudes are visible; larger spikes are from ab3A neuron while smaller spikes are from ab3B neuron. Top two recordings are from parental line *w*; ∆*halo*/*CyO*; *DmelOr22a-Gal4* with native expression of DmelOR22a in ab3A. The bottom five recordings are from test flies ∆*halo*; *DmelOr22a-Gal4*/*UAS-ORX* with heterologous expression of moth ORs in ab3A.

Table S1. List of odorants.

Chemical class	Odor name	CAS number
aromatic	2-phenyl ethanol	60-12-8
	benzyl acetone	2550-26-7
	p-cresol	106-44-5
	acetophenone	98-86-2
	veratrole	91-16-7
	benzyl acetate	140-11-4
	methyl benzoate	93-58-3
	p-toluquinone*	553-97-9
	phenyl acetaldehyde	122-78-1
	4-ethyl guaiacol	2785-89-9
	benzyl alcohol	100-51-6
	benzaldehyde	100-52-7
	cinnamaldehyde	104-55-2
	methyl salicylate	119-36-8
	eugenol	97-53-0
	DEET	134-62-3
	Z3-hexenyl benzoate	25152-85-6
	benzyl salicylate	118-58-1
	benzoic acid*	65-85-0
terpene	(+)-linalool	126-90-9
	(-)-linalool	126-91-0
	sulcatone	110-93-0
	geraniol	106-24-1
	nerol	106-25-2
	(±)-linalool	78-70-6
	valencene	4630-07-3
	Z-verbenol*	18881-04-4
	(-)-menthone	14073-97-3
	carvacrol	499-75-2
	(+)-limonene	5989-27-5
	eucalyptol	470-82-6
	geranyl acetate	105-87-3
	β-caryophyllene	87-44-5
	β-ocimene	13877-91-3
	E-nerolidol	40716-66-3
	α-farnesene	502-61-4
	β-myrcene	123-35-3
acid	heptanoic acid	111-14-8
	hexanoic acid	142-62-1
	butyric acid	107-92-6

	acetic acid	64-19-7
	octanoic acid	124-07-2
	2-oxopentanoic acid	1821-02-9
	propanoic acid**	79-09-4
alcohol	1-octen-3-ol	3391-86-4
	hexanol	111-27-3
	Z3-hexenol	928-96-1
	octanol	111-87-5
	methionol	505-10-2
	nonanol	143-08-8
aldehyde	E2-hexenal	6728-26-3
	hexanal	66-25-1
	nonanal	124-19-6
	octanal	124-13-0
	decanal	112-31-2
ester	ethyl hexanoate	123-66-0
	Z3-hexenyl acetate	3681-71-8
	methyl hexanoate	106-70-7
	ethyl tiglate	5837-78-5
	E2-hexenyl acetate	2497-18-9
	Z3-hexenyl propionate	33467-74-2
	butyl butyrate	109-21-7
	propyl valerate	141-06-0
	ethyl sorbate*	2396-84-1
ketone	Z-jasmone	488-10-8
	2-octanone	111-13-7
	2-heptanone	110-43-0
	γ-hexalactone	695-06-7
	2-hexanone	591-78-6
	2,3-butanedione	431-03-8
	2-nonanone	821-55-6
nitrogenous	3-methylbutyl aldoxime	(E/Z) 5775-74-6 / 5780-40-5
	methyl anthranilate	134-20-3
	2-acetyl pyridine	1122-62-9
	2-methylbutyl aldoxime	(E/Z) 49805-55-2 / 49805-56-3
	ethyl anthranilate	87-25-2
	nicotine	54-11-5
	indole	120-72-9
	pyrrolidine	123-75-1
	cadaverine**	462-94-2

The solvent was hexane or acetone (*) or distilled H_2O (**).

Table S2. List of primers.

	Forward primer	Reverse primer
Integration vector primer, pUASinsert	ACTACTGAAATCTGCCAAGAAGT	CCTTAGAGCTTTAAATCTCTGTAGG
Genotyping primer target 1: AAAACTCTTACAGGAAAACA	CGATTAAGTGCCAGTTTCACAA	ATACATGCGGTAAGTCTTGGCT
Genotyping primer target 2: AAGATACAGAAAACGAAGTC	CCACATAGATCAAAATTGGGGT	CCGTTATGACGTGTTCAATGTC
RT-PCR primer, MsexOR36 CDS	ATGGCCGAAACTCTTTTAGACAAATCG	TTACAAGGAAGTTGGGCACGAGTT

Table S3. Correlation imaging results versus SSR. (Related to Figure 3)

	glomerulus	1	2	3	4	5	6	7	8	9	10	11	12	13	14	15	16	17	18	19	20	21	22	23
**MsexOR80**	Spearman's r	0.38	0.40	0.29	0.37	0.33	0.48	0.30	0.19	0.19	0.21	0.27	0.56	0.27	0.14	0.19	0.14	0.13	0.26	0.20	0.08	0.13	-0.01	0.09
	p-value	0.0005	0.0003	0.0085	0.0008	0.0025	< 0.0001	0.0063	0.0871	0.0863	0.0664	0.0155	< 0.0001	0.0139	0.1998	0.0974	0.2023	0.2394	0.0182	0.0781	0.4994	0.2447	0.8956	0.41
**MsexOR33**	Spearman's r	0.35	0.33	0.27	0.37	0.31	0.49	0.28	0.12	0.13	0.14	0.24	0.57	0.26	0.12	0.14	0.14	0.10	0.20	0.14	0.00	0.09	0.01	0.05
	p-value	0.0016	0.0027	0.014	0.0008	0.0055	< 0.0001	0.011	0.2823	0.2477	0.2066	0.0314	< 0.0001	0.02	0.2772	0.2134	0.2092	0.3553	0.0733	0.2324	0.9773	0.4401	0.903	0.6853
**MsexOR8**	Spearman's r	0.34	0.31	0.22	0.23	0.24	0.41	0.25	0.07	0.17	0.22	0.28	0.58	0.15	-0.04	-0.03	0.01	-0.07	0.17	0.07	0.04	0.12	-0.07	-0.07
	p-value	0.002	0.0047	0.0534	0.0395	0.0292	0.0001	0.027	0.5353	0.1376	0.0499	0.0132	< 0.0001	0.1719	0.7455	0.8116	0.9614	0.5301	0.1422	0.522	0.7332	0.3094	0.5309	0.5502
**MsexOR36**	Spearman's r	0.27	0.31	0.22	0.29	0.21	0.44	0.19	0.15	0.13	0.13	0.13	0.67	0.22	0.11	0.13	0.13	0.14	0.23	0.13	0.06	0.11	-0.16	-0.01
	p-value	0.0171	0.0047	0.0543	0.0102	0.056	< 0.0001	0.0855	0.1859	0.2495	0.2574	0.2426	< 0.0001	0.0537	0.3191	0.2413	0.266	0.2014	0.0364	0.2506	0.6187	0.3221	0.145	0.9292

significant after Holm-Bonferroni correction for multiple comparisons

Table S4. Imaging results of glomerulus 12 to nonanal and propyl valerate. (Related to Figure 3)

glom#12 (deltaF/F)

	**nonanal**				**propyl valerate**		
	10^-5	10^-4	10^-3		10^-5	10^-4	10^-3
	0.1372	0.1386	0.3516		0.1771	0.5607	0.5718
	0.0366	0.0989	0.8924		1.4001	1.4151	1.4653
	0.2336	0.2219	0.6444		0.9049	1.6609	1.2232
	0.3246	0.2031	0.0978		0.2504	1.5307	1.6612
	0.5109	1.0389	0.7260		1.1666	1.6573	1.7164
	0.1194	0.3263	0.7020		0.8671	0.7642	1.1671
	-0.0105	0.0617	0.0672		0.4455	0.7324	1.0219
	0.1917	0.0709			0.9583	1.4836	1.6376
	0.1832				0.4946	0.5743	1.4402
					0.2562	1.0953	0.9130
					1.0209		0.9560
**median**	**0.18**	**0.17**	**0.64**		**0.87**	**1.26**	**1.22**

Table S5. Imaging results of glomerulus 12 of heterozygous versus homozygous MsexOR36 mutant moths. (Related to Figure 4)

glomerulus 12 (deltaF/F)

***MsexOR36* (+/-)**	benzyl acetate	benzyl acetone	linalool	sulcatone	β-ocimene	1-octen-3-ol	butyl butyrate	ethyl hexanoate	propyl valerate	γ-hexalactone	2-nonanone	2-octanone
Heterozygous_1	1.015	1.060	1.106	0.762	0.748	1.081	0.955	1.558	0.698	0.969	0.809	0.994
Heterozygous_2	0.407	0.915	0.402	0.533	0.000	0.599	0.135	0.577	0.407	0.559	0.660	0.733
Heterozygous_3	0.668	0.783	0.701	0.803	1.187	1.361	1.293	1.187	1.080	0.841	0.795	0.837
Heterozygous_4	1.255	1.264	1.012	0.847	0.662	1.518	1.084	1.136	0.362	1.117	1.290	0.925
Heterozygous_5	0.461	0.270	0.552	0.573	0.740	0.846	0.867	0.708	0.593	0.578	0.782	0.578
Heterozygous_6	1.220	0.852	1.155	1.006	1.299	1.623	1.450	1.382	1.536	1.225	1.410	1.144
Heterozygous_7	0.904	0.490	0.533	0.764	0.217	1.262	0.972	1.083	0.886	1.159	0.877	0.592
Heterozygous_8	0.914	0.578	0.612	1.085	1.088	1.457	1.594	0.669	0.706	0.668	0.675	0.667
**median**	**0.91**	**0.82**	**0.66**	**0.78**	**0.74**	**1.31**	**1.03**	**1.11**	**0.70**	**0.91**	**0.80**	**0.79**
***MsexOR36* (-/-)**												
homozygous_1	0.702	0.727	0.491	1.245	0.061	1.074	0.787	0.961	0.973	1.079	0.971	0.942
homozygous_2	1.253	0.817	0.768	0.929	0.039	1.372	0.916	0.832	0.607	1.088	0.804	0.444
homozygous_3	0.596	0.141	0.420	0.201	0.165	0.642	0.858	0.954	0.385	0.698	0.442	0.632
homozygous_4	1.183	0.473	0.254	0.761	0.278	1.147	1.053	0.868	1.274	1.230	1.107	0.634
homozygous_5	0.497	1.044	0.290	0.822	0.000	1.034	0.514	1.133	0.528	0.359	0.418	0.567
homozygous_6	0.997	1.147	1.102	0.747	0.143	1.479	0.894	1.109	0.873	1.058	0.956	0.966
homozygous_7	0.955	0.627	0.550	0.926	0.000	0.864	1.096	0.713	1.127	1.043	0.435	0.793
homozygous_8	1.208	1.012	1.292	1.411	0.027	2.009	1.464	1.655	1.995	1.157	1.462	1.704
homozygous_9	2.087	0.933	0.994	0.729	0.000	1.244	1.154	1.537	1.293	0.991	0.908	1.238
homozygous_10	0.686	0.593	0.456	1.338	0.000	1.310	1.289	0.625	0.925	0.490	0.515	0.681
**median**	**0.98**	**0.77**	**0.52**	**0.87**	**0.03**	**1.20**	**0.98**	**0.96**	**0.95**	**1.05**	**0.86**	**0.74**
Mann-Whitney U test												
p=	0.4807	0.9654	0.3599	0.5726	0.0062	0.7618	0.5726	0.9999	0.3154	0.8968	0.7618	0.9654

References

1. Carey, A.F., Wang, G.R., Su, C.Y., Zwiebel, L.J., and Carlson, J.R. (2010). Odorant reception in the malaria mosquito *Anopheles gambiae*. Nature *464*, 66-77.
2. Grabe, V., Baschwitz, A., Dweck, H.K.M., Lavista-Llanos, S., Hansson, B.S., and Sachse, S. (2016). Elucidating the neuronal architecture of olfactory glomeruli in the *Drosophila* antennal lobe. Cell Reports *16*, 3401-3413. 10.1016/j.celrep.2016.08.063.
3. de Fouchier, A., Walker, W.B., Montagne, N., Steiner, C., Binyameen, M., Schlyter, F., Chertemps, T., Maria, A., Francois, M.C., Monsempes, C., et al. (2017). Functional evolution of Lepidoptera olfactory receptors revealed by deorphanization of a moth repertoire. Nature Communications *8*, 15709. 10.1038/ncomms15709.
4. Chang, H.T., Unni, A.P., Tom, M.T., Cao, Q., Liu, Y., Wang, G.R., Llorca, L.C., Brase, S., Bucks, S., Weniger, K., et al. (2023). Odorant detection in a locust exhibits unusually low redundancy. Current Biology *33*. 10.1016/j.cub.2023.11.017.
5. del Mármol, J., Yedlin, M.A., and Ruta, V. (2021). The structural basis of odorant recognition in insect olfactory receptors. Nature *597*, 126-131. 10.1038/s41586-021-03794-8.
